# Chlorophyll excitation energies and structural stability of the CP47 antenna of photosystem II: a case study in the first-principles simulation of light-harvesting complexes†

**DOI:** 10.1039/d0sc06616h

**Published:** 2021-02-12

**Authors:** Abhishek Sirohiwal, Frank Neese, Dimitrios A. Pantazis

**Affiliations:** Max-Planck-Institut für Kohlenforschung Kaiser-Wilhelm-Platz 1 45470 Mülheim an der Ruhr Germany; Fakultät für Chemie und Biochemie, Ruhr-Universität Bochum 44780 Bochum Germany dimitrios.pantazis@kofo.mpg.de

## Abstract

Natural photosynthesis relies on light harvesting and excitation energy transfer by specialized pigment–protein complexes. Their structure and the electronic properties of the embedded chromophores define the mechanisms of energy transfer. An important example of a pigment–protein complex is CP47, one of the integral antennae of the oxygen-evolving photosystem II (PSII) that is responsible for efficient excitation energy transfer to the PSII reaction center. The charge-transfer excitation induced among coupled reaction center chromophores resolves into charge separation that initiates the electron transfer cascade driving oxygenic photosynthesis. Mapping the distribution of site energies among the 16 chlorophyll molecules of CP47 is essential for understanding excitation energy transfer and overall antenna function. In this work, we demonstrate a multiscale quantum mechanics/molecular mechanics (QM/MM) approach utilizing full time-dependent density functional theory with modern range-separated functionals to compute for the first time the excitation energies of all CP47 chlorophylls in a complete membrane-embedded cyanobacterial PSII dimer. The results quantify the electrostatic effect of the protein on the site energies of CP47 chlorophylls, providing a high-level quantum chemical excitation profile of CP47 within a complete computational model of “near-native” cyanobacterial PSII. The ranking of site energies and the identity of the most red-shifted chlorophylls (B3, followed by B1) differ from previous hypotheses in the literature and provide an alternative basis for evaluating past approaches and semiempirically fitted sets. Given that a lot of experimental studies on CP47 and other light-harvesting complexes utilize extracted samples, we employ molecular dynamics simulations of isolated CP47 to identify which parts of the polypeptide are most destabilized and which pigments are most perturbed when the antenna complex is extracted from PSII. We demonstrate that large parts of the isolated complex rapidly refold to non-native conformations and that certain pigments (such as chlorophyll B1 and β-carotene h1) are so destabilized that they are probably lost upon extraction of CP47 from PSII. The results suggest that the properties of isolated CP47 are not representative of the native complexed antenna. The insights obtained from CP47 are generalizable, with important implications for the information content of experimental studies on biological light-harvesting antenna systems.

## Abstract 2

Advanced QM/MM simulations explore the excited states of a photosynthetic light-harvesting antenna in its physiologically complexed state and model the consequences of extraction on conformational and electronic properties.

## Graphical abstract

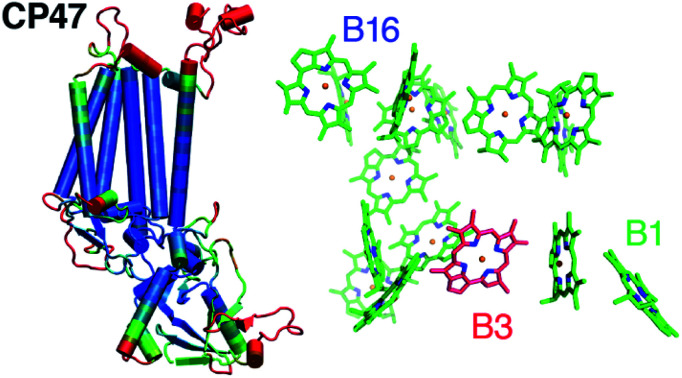

## 1. Introduction

Light harvesting and excitation energy transfer are essential processes in natural photosynthesis, performed by arrays of chromophores embedded in functional protein matrices.^1–3^ In the case of the enzyme photosystem II (PSII) of oxygenic photosynthesis these processes involve both extrinsic antenna proteins, such as peripheral light harvesting complexes, and intrinsic antenna proteins that are integral components of a functional PSII assembly.^1^ These core antenna proteins in PSII are the transmembrane chlorophyll-binding proteins CP43 (PsbC) and CP47 (PsbB) (*ca.* 43 and 47 kDa respectively), which contain chlorophyll *a* and carotenoid molecules. The core antenna proteins interact closely with the reaction center proteins D1 and D2, which host the chromophores responsible for converting the excitation energy to charge separation, driving water oxidation and plastoquinone reduction in PSII.^4–7^ CP43 and CP47 absorb light and deliver excitation energy to the reaction center, but they also accept and relay excitation energy to the reaction center from extrinsic light-harvesting antennae.^8–15^ In addition, they maintain the structural integrity of the reaction center and even, in the case of CP43, participate in ligation and stabilization of the all-important oxygen–evolving complex.^16–18^

**Fig. 1:**
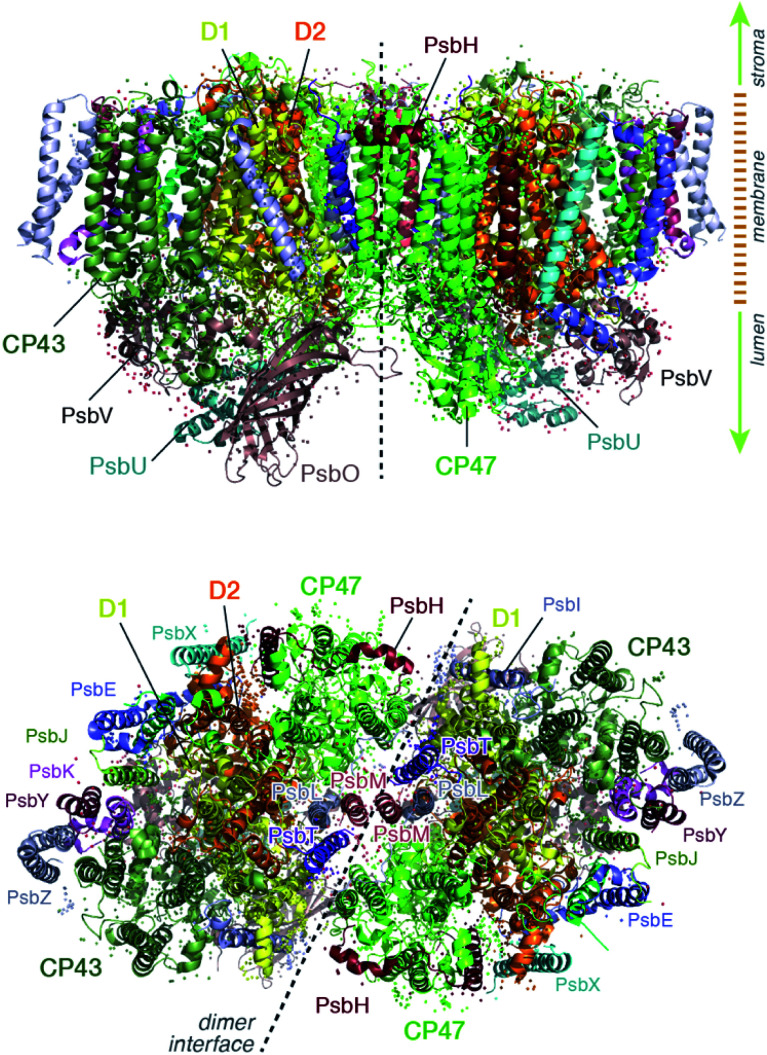
Overall structure of the photosystem II dimer. View from the side (along the membrane) and from the stromal side of the membrane (the acceptor side of PSII), indicating the location of the CP47 and CP43 core antenna complexes along with other key proximal proteins.

The position of CP43 and CP47 within the PSII dimer is indicated in Fig. 1. Both pigment–protein complexes contain six transmembrane helices arranged in pairs. In the available structural models of PSII, CP43 binds 13 chlorophyll molecules and CP47 binds 16 chlorophyll molecules, as well as 3 and 4 carotenoids, respectively. CP43 is situated at the outer region of PSII and interacts with the D1 protein (PsbA), whereas CP47 is positioned at the interface between the two monomers of a PSII dimer. CP47 forms close contacts with the D2 protein (PsbD), as well as proteins H (PsbH), L (PsbL) and M (PsbM).

Knowledge of the site energies of individual chromophores and their excitonic couplings is essential for understanding the function of the intrinsic light harvesting systems, elucidating the interactions between the different components of PSII and the mechanism of excitation energy transfer to the reaction center. However, direct experimental determination of site energies is impossible, while indirect deduction through fitting of experimental data such as absorption, linear or circular dichroism and fluorescence is challenging due to multiple reasons: spectral congestion resulting from the presence of multiple chlorophylls with similar spectral properties, uncertainties in the fitting of experimental data and in the attribution of specific parameters to specific pigments, different origin of structural *versus* spectroscopic data, and variations in samples and preparations leading to variability in the primary data and not simply in their modelling and interpretation. Specifically for CP47 different groups have reached strongly conflicting conclusions regarding the chlorophyll site energies in general, the nature of low-energy states, and the identity of the lowest energy pigment(s) (or “red chlorophylls”) in particular, a key piece of information for determining the directionality and kinetics of energy transfer.^11–15,19–23^

**Fig. 2:**
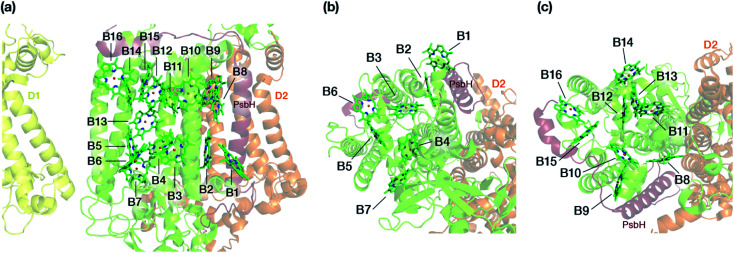
(a) The chlorophylls of the CP47 complex (green color), labelled according to the nomenclature proposed by Müh and Zouni.^1^ This side view is analogous to that of the top panel of Fig. 1. The PsbH and D2 polypeptides of the same PSII monomer as well as the D1 chain of the other monomer are also depicted for orientation. (b) View of chlorophylls B1–B7 from a lumenal perspective (bottom view with respect to panel a). (c) View of chlorophylls B8–B16 from a stromal perspective (top view with respect to panel a).

The chlorophyll molecules embedded in CP47 are depicted in Fig. 2. Different nomenclatures for these chlorophylls are used in the literature. In our study we adopt the nomenclature recently proposed by Müh and Zouni and we refer the reader to their review^1^ for additional details and discussion. Table 1 lists the “B*x*” (*x* = 1–16) labels used in the present work along with equivalent labels from widely used crystallographic models of PSII. The chlorophylls are seen to be distributed approximately in two layers within the transmembrane region and approximately parallel to the membrane plane, one layer closer to the stromal and one closer to the lumenal side, with B13 being somewhat in-between the two layers. This spatial arrangement contrasts with the arrangement of RC chromophores, which are distributed in two branches that run perpendicular to the membrane. No chlorophylls are embedded in the section of the CP47 polypeptide that protrudes into the lumen.

**Table 1:** Nomenclature for the CP47 chlorophylls. His_δ_ and His_ε_ denotes the binding mode of histidine residues with respect to the N_δ_ and N_ε_ position, respectively. Axial ligation on the opposite and the same side of the phytyl chain are denoted as α and β type ligation. All amino acid residues are from CP47 (PsbB) unless otherwise indicated

	2AXT^24^	3ARC^25^	3WU2 (ref. 25)^a^	Axial ligand^b^	Keto H-bond^c^	Comment
B1	11	612	604	H_2_O (α)	H_2_O (bulk)	At protein–bulk interface
B2	12	613	605	His_ε_201 (α)	—	Close contact with PsbH
B3	13	614	606	His_δ_202 (α)	Arg68	—
B4	14	615	607	His_δ_455 (α)	—	—
B5	15	616	608	His_δ_100 (α)		At dimer interface
B6	16	617	609	His_ε_157 (α)	—	At protein-bulk interface
B7	17	618	610	H_2_O (α)	Tyr40	At dimer interface
B8	21	619	611	His_δ_466 (α)	Ser240 & H_2_O	Close contact with D2
B9	22	620	612	His_δ_216 (α)	H-Thr27	Direct interaction with PsbH
B10	23	621	613	H_2_O (β)	His_δ_142	His_δ_142 axially ligated to B15
B11	24	622	614	His_δ_469 (α)	H_2_O	—
B12	25	623	615	His_δ_23 (β)	Ser241	—
B13	26	624	616	His_δ_26 (β)	—	At dimer interface
B14	27	625	617	His_δ_9 (α)	—	At dimer interface
B15	28	626	618	His_δ_142 (β)	His_δ_23	His_δ_23 axially ligated to B12
B16	29	627	619	His_δ_114 (β)	H-Thr5 & H_2_O (bulk)	At dimer–bulk interface, direct interaction with PsbH

a Supersedes 3ARC.

b Numbering based on 3WU2.

c The keto partner may differ in crystal structures obtained from different species.

The majority of chlorophylls (13 out of 16) have histidine residues as axial ligands, whereas the remaining three are ligated by a water molecule. Two classes can be distinguished in the histidine ligated chromophores, *i.e.* ligation with respect to the N_ε_ or N_δ_ position of the side chain imidazole. Overall, the axial ligations also differ in their respective mode, for example 11 out of 16 chlorophylls have α-type ligation, which means that the phytyl chain and the axial ligand are on opposite sides of the macrocyclic ring. In addition, the chromophores have different hydrogen bonding partners to the keto group, which may be derived from protein residues, special lipid molecules, or conserved or bulk water molecules. Overall, it is evident that the chlorophylls have both a distinct protein environment and a distinct binding mode to the protein.

The site energy of one of the chromophores of the CP47 antenna complex is believed to be lower than that of the initial electron donor in the PSII reaction center. In practice such chlorophyll could become the sink of excitation energy and inhibit further transfer to the reaction center, therefore impacting the overall excitation energy transfer kinetics of oxygenic photosynthesis. This low energy trap in CP47 can be identified from the fluorescence band feature at 695 nm,^26^ known as F695. Therefore, the identity of this particular chlorophyll is important for our overall understanding of the excitation energy transfer process from the CP47 to the reaction center. The identity of this chlorophyll has been debated over the years and has led to conflicting proposals, with no consensus emerging so far. A significant part of research work on the CP47 protein hints at B16 as the prime candidate for the low-energy trap. Spectroscopic measurements by D'Haene *et al.*^27^ using PSII samples lacking the psbH protein did not observe the F695 emission feature. Based on these results, B16 was proposed to be the long-wavelength chlorophyll, as it shares a direct hydrogen bonding interaction with Thr5 of PsbH. Similar hints were provided by Fluorescence Line Narrowing (FLN) spectra of de Weerd *et al.*,^23^ who proposed that the long-wavelength chlorophyll shares a strong hydrogen bond with the protein through the keto group. Site-directed mutagenesis studies^28^ performed by mutating His114 (axial ligand to B16) to a tyrosine led to the disappearance of the F695 feature in the fluorescence band as well as overall destabilization of photosystem II and reduction of its catalytic efficiency.^29^ Based on these results, B16 is suggested as the chlorophyll with the lowest site energy. A similar suggestion was made by Vasil'ev *et al.*^30,31^ Jankowiak and co-workers^21,22^ also proposed B16 to be a major contributor to the low-energy spectrum, however they also suggested that other possibilities are equally acceptable, such as B11 and B13. B13 was also proposed to be the red chlorophyll in a study by Reppert *et al.*^32^ Based on analysis and fitting of circular dichroism (CD) and circularly polarized luminescence (CPL) data, Hall *et al.*^19^ proposed B1 to possess the lowest site energy in CP47, whereas a study by Skandary *et al.*^33^ using single molecule spectroscopy (SMS) on monomeric and dimeric PSII found reduction in the F695 emission feature in the monomeric PSII and proposed B7 to carry the lowest site energy. The lack of consensus is abundantly clear.

Importantly, biophysical and spectroscopic characterizations are often performed on samples of isolated CP47, which means that most experimental studies are performed on entities separated from their PSII environment. Such treatments may result in the isolated complexes losing their “native” spectroscopic properties, especially in the case of reaction center complexes.^34,35^ Variation in the nature of different CP47 preparations may be a reason for the apparent incompatibility of different data sets. It is also likely that one or more chlorophylls may be lost under certain treatments. Therefore, it would be useful to have (a) a view of the site energies of the CP47 chlorophylls computed on the basis of a realistic model of a complete PSII dimer, and (b) information regarding the specific regions of CP47 that are most structurally destabilized upon isolation compared to the PSII-associated form. This work attempts to address both of the above points.

First, we employ a high-level quantum chemical method in the framework of a large-scale QM/MM approach to compute the low-energy excited state properties of all CP47 chromophores taking explicitly into account the protein–pigment interactions. The protein environment of the entire PSII dimer is explicitly considered at an all-atom level. We compare the dimeric PSII values with “gas phase” results, where the excited state energies are computed in the absence of the protein matrix. This gives us a clear view of how the anisotropic protein electrostatics affect the excitation profile of CP47. The second part of this study addresses the question of structural intactness of CP47 when it is extracted from PSII. In this case, molecular dynamics simulations of CP47 for a few hundred nanoseconds were carried out. The structural changes in these simulations are monitored and analyzed to identify the regions of the protein that are particularly susceptible to structural modifications and the specific chromophores that are mostly destabilized and might be severely perturbed or even lost upon extraction of CP47 compared to the natively assembled PSII system.

## 2. Methodology

### 2.1. Preparation of models

**Fig. 3:**
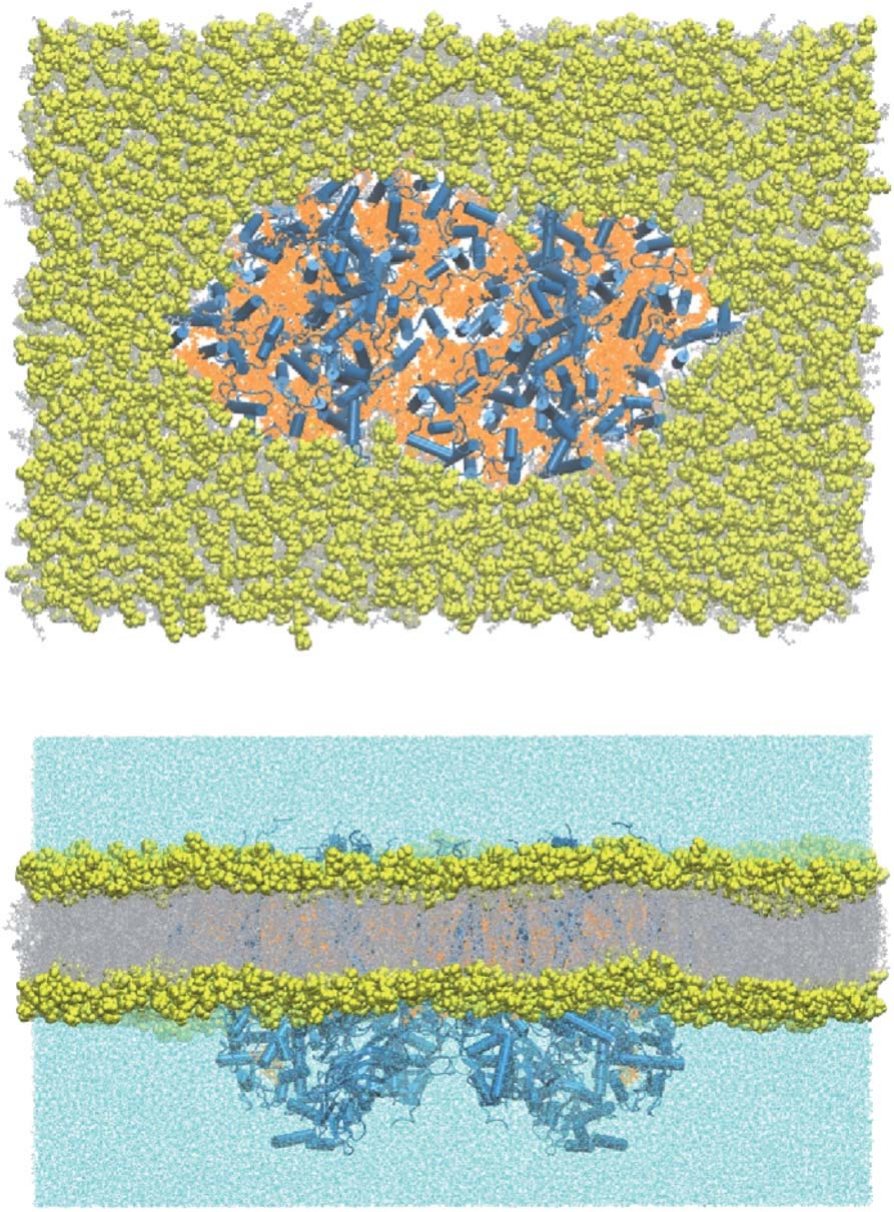
Top (stromal) and side view of the molecular mechanics model of the PSII dimer embedded in an equilibrated POPC lipid bilayer.

The lipid-bilayer embedded model of photosystem II is based on the high-resolution dimeric crystal structure (1.9 Å) of *Thermosynechococcus vulcanus* (PDB ID: 3WU2).^25^ In the present work, we have used the complete PSII dimer to build the entire molecular mechanics based model (Fig. 3). All water molecules resolved by X-ray crystallography were retained. The PSII dimer complex was embedded in a POPC (1-palmitoyl-2-oleoyl-*sn*-glycero-3-phosphocholine) lipid bilayer of dimensions 280 × 195 × 158 Å^3^ using the Packmol-Memgen.^36,37^ Appropriate amounts of Na^+^ and Cl^−^ ions were added to neutralize the system and maintain a physiological concentration of 0.15 M. The entire system consists of 881 026 atoms. The electrostatic charges for all the cofactors were computed based on the MK-RESP (Merz–Kollman Restrained Electrostatic Potential) methodology.^38^ Parameters for the standard protein residues were described using the Amber14SB force field^39^ and TIP3P model^40^ is chosen for the water. The bonded parameters for the organic cofactors were described using GAFF2.^41^ The LIPID17 force field^42,43^ was chosen to describe the POPC bilayer. For Na^+^ and Cl^−^ we used the Joung–Cheathem parameters compatible with the TIP3P water model.^44,45^

The molecular mechanics model for the isolated CP47 was prepared starting from the configuration in the crystal structure (PDB ID: 3WU2). All crystallographic waters belonging to the CP47 subunit were kept during the system preparation. The Complete isolated CP47 subunit was embedded inside a water box of dimension 135 × 135 × 135 Å^3^. Appropriate amounts of counter-ions were added to neutralize the system. The entire system contained 216 992 atoms. The force-field parameters for different components in this case are the same as described above for the lipid bilayer bound PSII dimer.

### 2.2 Classical molecular dynamics

The complete PSII lipid-bilayer system was minimized systematically in order to remove the unfavorable geometric clashes. During the equilibration phase, the system was slowly heated from 10 to 100 K within 5 ps (picoseconds) in the NVT ensemble. In the next step, the temperature was slowly increased from 100 to 303 K in the NPT ensemble for 100 ps, while maintaining the positional restraints (20 kcal mol^−1^ Å^−2^) on the C_α_ atom of amino acids. The temperature during this procedure was controlled using the Langevin dynamics^46^ with a collision frequency of 5 ps^−1^. The system was then further equilibrated for another 5 ns (nanoseconds) in the NPT ensemble with the same C_α_ restraints. During this step, the collision frequency (Langevin dynamics) was reduced to 1 ps^−1^ and the pressure was regulated anisotropically using the Berendsen barostat^47^ with a relaxation time of 2 ps, maintained at 1 bar. The particle Mesh Ewald (PME)^48^ approach was used to treat all electrostatic interactions, with a 10 Å cut-off. The bonds involving the hydrogen atoms were constrained using the SHAKE algorithm, therefore a time-step of 2 fs is applied throughout. The frames were saved every 2 ps.

The molecular mechanics set up for the isolated CP47 was thoroughly and systematically energy minimized to remove geometric clashes. The equilibration of the system was carried out in two steps. First, we increased the temperature slowly from 10 to 300 K within 1 ns in the NVT ensemble. The temperature during this procedure was controlled using the Langevin dynamics^46^ with a collision frequency of 5 ps^−1^. In the second step, we propagated the system for 5 ns in the NPT ensemble at 300 K. During the equilibration protocol, the C_α_ atoms of amino acids were restrained with a weight of 20 kcal mol^−1^ Å^−2^. After thorough equilibration, we initiated the production simulation for 200 ns in the NPT ensemble. For production simulations, the collision frequency (Langevin dynamics) was set to 1 ps^−1^ and the pressure was regulated isotropically using the Berendsen barostat^47^ with a relaxation time of 1 ps, maintained at 1 bar. The frames were saved every 10 ps. A time-step of 2 fs was applied in production simulations. All classical molecular dynamics simulations in this work were performed using the GPU enabled *pmemd* engine^49–51^ of the AMBER18 package.^52,53^ Analysis of the trajectories was performed using the CPPTRAJ^54,55^ module of AmberTools19.

### 2.3. QM/MM optimization protocol

The QM/MM computations on the CP47 chromophores were performed using the equilibrated PSII-lipid bilayer system. The snapshot for this task was derived after clustering^56^ the frames from the last 4 ns of the MD, using the CPPTRAJ^54,55^ module of AmberTools19. The DBSCAN clustering algorithm^57^ was used with minimum points set to 2 to form a cluster. The distance cut-off between points for cluster formation was set to 1. The RMS (root mean square) fit to the protein backbone atoms (*i.e.* C_α_ atoms and no hydrogen atoms) was used as a distance metric between frames during the clustering process. One populated cluster is obtained from the clustering protocol, which is used for the QM/MM computations. For the QM/MM setup, we considered the entire PSII dimer and a water shell (2 Å around the protein), which also includes all the waters present in the protein cavity and various channels. The final system used in the QM/MM calculations contains a total of 99 673 atoms. The QM/MM calculations were performed with the ChemShell 3.7 program,^58–60^ where the in-built DL-POLY^61^ was used for the MM computations and ORCA^62^ was used as the QM engine. The QM/MM calculations presented here are based on the electrostatic embedding technique.

In QM/MM geometry optimization the entire system is divided into two parts, active and static. In the present case the active part consists of the QM region and a surrounding MM environment (*ca.* 11 Å around the central magnesium of the chlorophyll pigment). The atoms in the active part, both QM and MM, are free to move during the geometry optimization step. The static part remains frozen throughout the geometry optimization process and only acts as an electrostatic environment. The QM region in each geometry optimization consists of the pigment with the axial ligand and the keto group hydrogen bonding partner (see also Table 1), but the phytyl chain is not included in the QM region due to negligible effect on Q-band energetics.^63^ The side-chains of protein residues acting as axial ligands and involved in hydrogen bonding with the keto group are included in the QM region. The atoms in the QM region connected with the active MM region through a covalent bond were cut using the hydrogen link atom technique. In case of the chlorophyll *a*, a link atom is placed between the C17^1^ and C17^2^ atoms. The charge-shift method implemented in ChemShell is used to avoid over-polarization of the QM region by the MM region. For all the geometry optimizations we employed the PBE functional^64^ and Def2-TVZP basis-sets,^65^ along with D3(BJ) dispersion corrections.^66,67^ To speed up the calculations of Coulomb integrals we employed the resolution of identity (RI) approximation,^68,69^ in combination with Weigend's universal Def2/J auxiliary basis set.^70^ We used tighter convergence criteria (for both geometry and SCF) and a higher DFT integration grid (grid6 in ORCA nomenclature) throughout the optimization protocol.

### 2.4. Computation of excitation energies

The computation of vertical excitation energies (VEE) in this work is performed on the QM/MM optimized geometries of the chromophores. The explicit effect of the entire PSII dimer environment on the electronic properties of chromophores is incorporated through point charges. Many of the CP47 chromophores lie on the contact point of the protein, membrane and bulk water, as a result the point-charge description of the membrane and bulk-water is also accounted for during these calculations. We used full Time-Dependent Density Functional Theory (TD-DFT), *i.e.* without applying the Tamm–Dancoff approximation, to compute the excited state properties using the range-separated *ω*B97X-V ^71–73^ functional with the Def2-TZVP basis set. The choice of this functional is motivated from successful application to the study of the PSII reaction center.^74^ The RIJCOSX approximation^75,76^ was used to speed up the calculations. In addition, very tight SCF convergence criteria were set along with higher DFT integration grids (grid6 and gridX7 in ORCA nomenclature) in all cases considered in this work.

## 3. Results and discussion

### 3.1. Chlorophyll site energies of complexed CP47

The energies of the lowest excited states of the CP47 chromophores are key ingredients in determining the characteristics of the excitation energy transfer process. Usually this critical piece of information is derived from spectral fitting that may partially take advantage of computationally derived parameters. Moreover, the experiments are generally performed on isolated CP47, *i.e.* in the absence of the native protein matrix. A tool that can provide complementary information and may circumvent these issues is the use of quantum chemical methods coupled with explicit consideration of protein environment effects through a QM/MM approach. The protein matrix is known to fine tune the properties of chromophores first of all through axial ligation, hydrogen bonding, and distortion of the macrocyclic ring. Once these structural effects are taken into account, protein electrostatics are the leading factor in modulating and differentiating the excited state properties of otherwise identical pigments. Here we report the site-energies of CP47 chlorophylls using a combination of state-of-the-art quantum chemical approaches within a QM/MM framework for the complete membrane-embedded dimeric PSII.

The geometries of all individual chromophores were optimized in a QM/MM approach as described in the methodology section. Subsequently, we proceeded with full TD-DFT calculations of site energies in three steps. First, we computed the site energy of CP47 chlorophylls in the gas phase using the QM/MM optimized geometries. We found that the computed site energies fall within the range of 1.888–1.981 eV (see Table 2). This demonstrates that the internal geometry of chromophores itself, for example *via* the steric control of the protein over macrocyclic ring curvature, is an important factor in differentiating the site energies. However, in the protein environment (using the electrostatic embedding technique) the site energies of the chromophores are further modulated, resulting in red or blue shifting relative to their gas-phase values (Table 2). This confirms the important electrostatic control of the protein matrix. We note that this type of control is not merely an additional perturbation but the dominant factor in determining the excitation profile and charge-transfer states in the case of the reaction center of photosystem II.^74^

**Table 2:** Computed site energies (in eV) of CP47 chromophores using the *ω*B97X-V functional with the Def2-TZVP basis set. The geometries in all cases are derived from QM/MM optimisations inside the complete PSII dimer. Parentheses indicate oscillator strengths

Site	*Q*_*y*_ a (vacuum)	*Q*_*y*_ (protein)	*Q*_*x*_ (protein)	*Q*_*x*_–*Q*_*y*_ (protein)
B1	1.941(0.22)	1.899 (0.25)	2.417 (0.06)	0.518
B2	1.928 (0.22)	1.953 (0.17)	2.374 (0.07)	0.421
B3	1.888 (0.23)	1.884 (0.19)	2.302 (0.01)	0.418
B4	1.935 (0.23)	1.947 (0.21)	2.459 (0.07)	0.512
B5	1.909 (0.22)	1.921 (0.17)	2.314 (0.01)	0.393
B6	1.924 (0.21)	1.933 (0.19)	2.390 (0.04)	0.457
B7	1.925 (0.23)	1.915 (0.21)	2.323 (0.03)	0.408
B8	1.951 (0.21)	1.967 (0.23)	2.436 (0.10)	0.469
B9	1.981 (0.22)	2.102 (0.20)	2.590 (0.31)	0.488
B10	1.965 (0.21)	1.966 (0.20)	2.430 (0.10)	0.464
B11	1.967 (0.23)	1.996 (0.24)	2.475 (0.14)	0.479
B12	1.939 (0.21)	1.941 (0.20)	2.413 (0.07)	0.472
B13	1.917 (0.23)	1.921 (0.27)	2.481 (0.10)	0.560
B14	1.942 (0.22)	1.956 (0.24)	2.428 (0.08)	0.472
B15	1.933 (0.22)	1.930 (0.21)	2.386 (0.06)	0.456
B16	1.945 (0.22)	2.050 (0.22)	2.488 (0.33)	0.438

a Single-point TD-DFT calculation performed on the QM/MM optimized geometry but without the point charge field of the protein.

Before discussing the results, we note that although previous studies have shown that the present methodology is sufficiently reliable to capture the electrostatic effect of the protein matrix and to reproduce the lowest excited states of chlorophylls with reasonable accuracy,^74^ we do not assert that the values reported here must be correct in an absolute numerical sense. Indeed, a detailed comparison of TD-DFT to wave function based correlated calculations at the coupled cluster level for gas-phase chlorophyll *a* suggested that TD-DFT values even with the best range-separated functionals may be blue-shifted.^63^ This probably also occurs here to some extent. Moreover, the values reported in Table 2 are vertical excitation energies. Nevertheless, the prediction of the ordering of site energies (relative differences) is expected to be robust for a given QM/MM optimized structural configuration. In addition to an inherent uncertainty related to the specific DFT approach, variation in absolute site energies and relative ordering may be observed with the dynamics of the pigment and protein matrix, as reported in a previous study of the PSII RC^74^ that employed the same methodology as the present work. For example, Chl_D1_ exhibited a minimum and maximum site energy of 1.85 and 1.88 eV (based on five snapshots with a 4 nanosecond interval),^74^ therefore, an uncertainty of *ca.* 30 meV may be presumed for the site energy of this pigment due to dynamics. Similar variations (ranging from *ca.* 5 to 35 meV) were observed for other RC pigments.^74^ Note that all RC pigments are deeply buried within the protein matrix similarly to the internal CP47 chlorophylls, therefore similar uncertainties in site energies can be assumed in the present case, whereas pigments such as B1 and B6 that are in close contact with the bulk are expected to have higher variation.

The present results indicate that within the CP47 protein chromophores B3 and B1 (both at the “bottom”/lumenal layer, see Fig. 2) are the most red-shifted, whereas B16 and B9 (both at the “top”/stromal layer) are the most blue-shifted. To the best of our knowledge, the possibility of B3 being the most red-shifted chromophore of CP47 has never been considered before. However, the second red-most chlorophyll identified here, *i.e.* B1, was most recently proposed by Hall *et al.*^19^ as a candidate for the lowest site energy chlorophyll of CP47. It is worth pointing out that of the B1 and B3 chromophores, B1 is at the edge of the protein-membrane-bulk water region. Possibly due to this reason it has the largest recorded crystallographic *B*-factor in the crystal structure.^25^ Due to its structurally less stable location and interaction with the bulk, coupled with the large internal geometry fluctuations due to protein dynamics, it is expected to have a broad distribution of site energy, as also pointed out by Hall *et al.*^19^ Similarly, a molecular dynamics study by Narzi *et al.*^77^ found that B1 has the highest atomic fluctuations among all CP47 chlorophylls. On the other hand, B3 is situated more deeply inside the CP47 protein matrix, and as a result large changes in its internal geometry are not expected. Among B1 and B3, the possibility of B3 being the lowest site energy chlorophyll is consistent with the work by Grondelle and co-workers,^23^ who proposed that the lowest site energy pigments share a strong hydrogen bond with the protein, in this case Arg68. On the other hand, the proposal of B1 is consistent with the experimental suggestion^26,78,79^ that the angle between the transition dipole moment of the low-energy state of CP47 and the thylakoid membrane is >35° in PSII core complexes. Overall, based on the intrinsic uncertainty in the computed site energies as estimated above, B1 and B3 can be considered approximately equally red-shifted. It is also hard to rule out chlorophyll B7, with the third lowest site energy in the current data set, as a candidate for the red chlorophyll.

The result on B16 is rather unexpected because B16 was long thought to be a prime candidate for the chlorophyll with the lowest site-energy. In a recent work^27^ conducted on the psbH removed PSII samples, it was found that the F695 emission feature was missing, and therefore it was concluded that B16 probably is the red chlorophyll, since it shares a hydrogen bond with the Thr5 of psbH. As a matter of fact, both B9 and B16 share hydrogen bonding interaction with the psbH protein. Similar conclusions were drawn by Jankowiak and co-workers,^20–22^ who proposed that B16 can be one of the major contributors to the low-energy trap. Similar observations were reported by Raszewski *et al.*,^13^ Shibata *et al.*,^12^ Polívka *et al.*^80^ and Mohamed *et al.*^81^ where B16 was proposed to have the lowest site energy.

The results presented in Table 2 are not directly comparable to site energy values obtained by fundamentally different approaches that involve fitting to various experimental data sets. However, we can compare the *relative* differences in site energies referenced to the chlorophyll with the lowest site energy. Fig. S1† provides such comparison of the relative site energies obtained in this work with previously published fitted values by Hall *et al.*^19^ (where B1 has the lowest site energy), by Shibata *et al.*^12^ (where B16 has the lowest site energy), and by Reinot *et al.*^21^ In the latter case we reproduce three of the reported sets, which are labelled “24A”, “26A”, and “29A” (lowest site energy at B11, B13, and B16, respectively).^21^ The comparison shows that no direct correlations can be made, neither between the present values and any of the fitted sets, nor between any of the fitted sets themselves. All relative site energies are within the 100 meV range for all fitted sets. The range spanned by the computed values for the relative site energies is compatible with this, with the exception of the blue-shifted B9 and B16 chlorophylls that fall outside the simulation envelope. This result might be related to the mixing of *Q*_*x*_ and *Q*_*y*_ states predicted by TD-DFT for these two chlorophylls (see below). Overall, the attribution of the lowest site energy chlorophyll appears closer to the conclusions of Hall *et al.* regarding the B1 site, but any further detailed one-to-one comparisons are not possible.

**Fig. 4:**
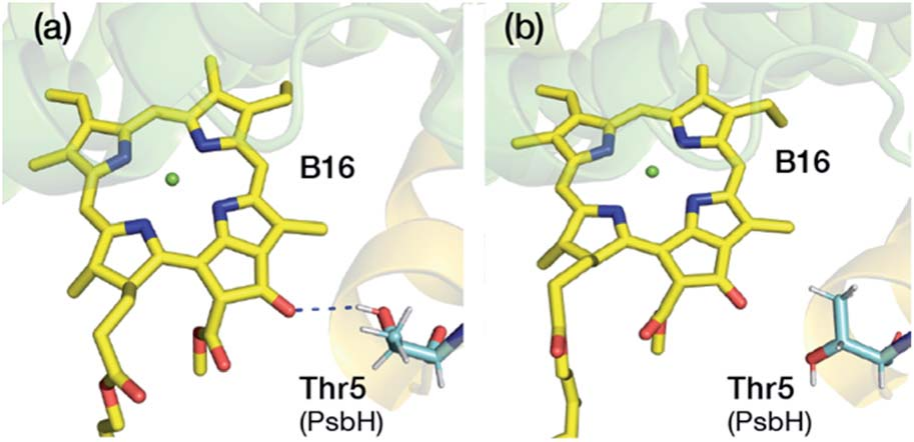
Depiction of two different conformations with (a) and without (b) hydrogen bond between the Thr5 of PsbH and the keto group of B16. In both cases B16, the axial ligand His114 and PsbH-Thr 5 were fully optimized in the QM/MM framework. The computed site energy of B16 without the H-bond is blue-shifted by 25 meV.

A lot of speculation exists^21,22,27^ in the literature regarding the role of the hydrogen bond between PsbH-Thr5 and B16 in modulating its site energy and introducing the F695 emission. Disregarding for a moment the result of the present study that B16 is a most unlikely candidate for the red chlorophyll, we tested the hypothesis that the hydrogen bond between Thr5 and B16 may introduce a red-shift in *Q*_*y*_ by performing geometry optimization of two different conformations of the threonine side chain, with and without the hydrogen bond with B16 keto-group. We found that there is no drastic change in the first excitation energy of B16 in the two models (Fig. 4). In fact, the removal of the hydrogen bonding to the keto group causes a red shift of less than 0.025 eV, rather than a blue shift, in the site energy of B16. Therefore, it is uncertain whether changes in the hydrogen bonding interaction between B16 and PsbH are in principle capable of introducing significant spectral changes by themselves, *i.e.* in the absence of additional, more significant, and not necessarily localized perturbations, or whether the direction of such local changes can be straightforwardly anticipated. We find it more likely that psbH loss triggers a series of structural alterations in addition to the simple removal of specific hydrogen bonds, that lead collectively to loss of the F695 emission feature. In this scenario, psbH removal does not red-shift chromophores through loss of specific hydrogen bonds but through more extensive unspecific modifications of the CP47 matrix.

Skandary *et al.*^33^ observed that monomeric PSII contains a smaller amount of red chlorophyll compared to dimeric PSII due to loss of the F695 emission feature. This points to structural reorganization of the monomeric unit and possible loss of cofactors at the dimer interface, suggesting a link between PSII dimerization and efficient light harvesting. It was speculated that the red chlorophyll(s) are situated on the PSII dimer interface, and that B7 is a candidate for the red trap. Since the structural changes between PSII monomer and dimer are ill-defined, we cannot ascertain the structure–property correlation with regard to F695. However, if we assume that the PSII monomer in this study contains intact PsbH, the results are in line with the preceding discussion, *i.e.* the absence of F695 does not have to correlate with removal of PsbH, and hence that the latter does not contribute to red trap formation through specific hydrogen bonding.

Hall *et al.* studied the F695 emission feature from samples containing isolated CP47 (from spinach). It was shown that the site energy of one or more chlorophylls would be as low as 1.80 eV. However, it is important to point out that the site energies reported in that work correspond to the absorption maximum, not the vertical excitation energies that we compute here. Based on our recent study on isolated Chl *a*,^63^ “quasi-experimental” vertical excitation energies for the *Q*_*y*_ band can be derived by empirically adding 50 meV to the band maximum. This would give us a “quasi-experimental” vertical excitation energy for the red-chlorophyll of *ca.* 1.85 eV. Keeping in mind a qualitative uncertainty estimate of *ca.* 0.03 eV, the vertical excitation energy of both B3 (1.884 eV) and B1 (1.899 eV) are in close agreement with the “quasi-experimental” vertical excitation energy. Therefore, it might be conjectured that B3 and/or B1 are responsible for the F695 emission. However, there are additional considerations regarding the existence of red chlorophyll(s) that will be discussed in the following.

Table 2 provides information on another aspect of the electronic structure, the second excited state *Q*_*x*_ and the gap between the first and second excited states. Unlike other light-harvesting chromophores, Chl *a* has smaller energy gap between *Q*_*y*_ and *Q*_*x*_ and as a result they may mix^63,82^ affecting exciton transport properties. The gap between *Q*_*y*_ and *Q*_*x*_ is modulated by both intrinsic (macrocyclic ring curvature) and extrinsic factors (such as axial ligand,^83^ hydrogen bonds, protein electrostatics *etc.*). Since each of the CP47 chromophores binds uniquely to the protein matrix it is expected that they have different *Q*_*y*_–*Q*_*x*_ energy gaps (Table 2). We found that B5 and B13 have the smallest and the largest *Q*_*y*_–*Q*_*x*_ energy gaps, respectively. Out of the low site energy chromophores, B1 has 0.1 eV larger *Q*_*y*_–*Q*_*x*_ energy gap than that of the B3. Interestingly, the blue-shifted B9 and B16 chromophores have an unusually high oscillator strength for the second excited state (nominally *Q*_*x*_) compared to the first (nominally *Q*_*y*_). Detailed analysis of the TD-DFT results shows that the present calculations predict a mixed character for the two lowest excitations for B9 and B16. Generally, in case of chlorophyll *a*, the *Q*_*y*_ band is dominated by the HOMO → LUMO followed by HOMO − 1 → LUMO + 1 excitations, whereas the *Q*_*x*_ band is dominated by the HOMO − 1 → LUMO followed by HOMO → LUMO + 1 excitations.^63^ In B9 and B16 the second main contribution to the first excited state comes from HOMO − 1 → LUMO instead of HOMO − 1 → LUMO + 1. Similarly, the second main contribution to the second excited state derives from HOMO → LUMO excitation instead of HOMO → LUMO + 1. Excited state calculations performed without the protein point charges do not yield this mixing, which confirms that the protein point charge field rather than any aspect of the internal geometry of the chromophores is responsible for the mixing of the two lowest states. It is not possible to elucidate at this point what might be the actual significance of this mixed character of B9 and B16 for light harvesting and excitation energy transfer.

An important question regarding the mechanism of excitation energy transfer is whether the site energy of the red chlorophyll of CP47 is lower than that of the primary electron donor of the PSII reaction center. Based on calculated results at exactly the same level of theory and with the same QM/MM computational approach published recently on the reaction center of PSII,^74^ we can state that none of the lowest site energies of CP47 chromophores computed here are lower than the computed values for the primary electron donor of the PSII reaction center.^74^ Similar results were obtained by Hsieh *et al.*^84^ using the charge density coupled^85^ method applied on molecular dynamics trajectories. Nevertheless, the present results clearly suggest that the site energies of B1 and B3 are of the same order of magnitude as the lowest excitonic state in the reaction center. Past theoretical analysis suggests that the lowest state with excitonic character in the reaction center of PSII is formed between Chl_D1_ and Pheo_D1_ (an excitonic state mixed with charge-transfer state)^74^ and that its energy is variable (1.84–1.88 eV for the given level of theory) based on inspection of a few different protein structural configurations, depending on the flexibility of the protein matrix.^74^ Due to mixing of excitonic and CT states the overall oscillator strength decreases compared to a pure excitonic state, which might decrease the probability of efficient excitation energy transfer. Of course, these D1 pigments are much closer to the CP43 rather than the CP47 antenna. It thus remains an open question which RC chromophore first receives excitation energy from CP47. The D2 branch of the reaction center, consisting of Chl_D2_ and Pheo_D2_, is closer to the CP47 chromophores. Based on our previous study,^74^ Pheo_D2_ can be ruled as the initial excitation energy recipient from CP47 due to its blue-shifted *Q*_*y*_. Therefore, Chl_D2_ is the prime candidate for the early recipient of excitation energy from CP47. In addition, its lowest excitonic state is not coupled with any other reaction center chromophores. The calculated energy of its lowest excitonic state varies between 1.882–1.916 eV based on a few different protein configurations,^74^ which is of the same order or slightly blue-shifted compared to the site energies of B3 and B1 computed in the present work. Nevertheless, further studies need to be carried out in the future to understand the flow of energy between the antenna and reaction center and to understand if B3 and B1 are indeed traps for excitation energy transfer.

In summary, based on the results obtained in this work we rank B3 and B1 as the chromophores with the lowest site energies in “intact” CP47. Moreover, their computed site energies are of the same order of magnitude as the lowest excitonic state of the reaction centre simulated with the same theoretical approach. By contrast, B16, a widely proposed choice for the red chlorophyll, is computed to be one of the most blue shifted chlorophylls of CP47. The overall ranking of site energies produced by the present QM/MM calculations is distinct from any set previously discussed in the literature and as such it provides an alternative basis for modeling future experimental data obtained on maximally intact CP47 complexes consistent with the MM model employed in the present study.

### 3.2. Structural integrity of the isolated CP47 complex

A wide range of candidates for the red-trap in the CP47 were suggested from studies performed on isolated CP47 samples^19,86–91^ extracted/detached from PSII, since the study of intact complexed CP47 is extremely challenging due to significant contribution to absorption from the RC and CP43 chromophores. It is unknown to what degree the crystallographic structure of PSII-embedded CP47 remains consistent with the unknown structure of isolated CP47, or indeed if the isolated CP47 has a unique structure at all, adopts an undefined range of conformations, or maintains specific though non-native forms depending on the different extraction and sample preparation protocols. Ambiguity also persists on the presence of the psbH protein along with the isolated CP47. It is further speculated that some of the chlorophylls may be lost during such procedure. But even if the number of protein bound chlorophylls does not differ in the isolated *versus* intact CP47, it is unknown to what extent the site energies of the chromophores would be perturbed during isolation due to the unknown departure of the protein scaffold from its native complexed conformation and the loss of the native global electrostatics. In this section we try to approach some of the questions related to the structural aspects, in terms of protein flexibility and chromophore stability, of the isolated CP47 *versus* intact CP47 using all-atom classical molecular dynamics simulations. It is noted that the MD simulations were performed in water, *i.e.* no lipid bilayer or detergents were present, because our primary goal is to evaluate in the most direct way the main conformational changes of CP47 upon removal of the native environment. Our results will be presented for the protein overall, as well as for the chromophores specifically.

In our models we have maintained all chromophores belonging to intact CP47 (based on the 3WU2 PSII model) under the assumption that none of them are lost upon isolation. As a result, we have a total of 16 chlorophyll *a* and 4 β-carotene chromophores in our molecular mechanics model. The numbering scheme for the β-carotenes chromophores is adopted from Müh and Zouni, *i.e.* b1–b3 and h1.^1^ Two of the β-carotenes (b1 and b2) are situated right next to B14, whereas b3 is located close to B16. It is important to mention that the β-carotene which is close to chlorophyll B1 (h1) is either missing or modelled differently in different crystal structures, which is a strong indication of how fluxional it is, even in intact PSII sample preparations.

**Fig. 5:**
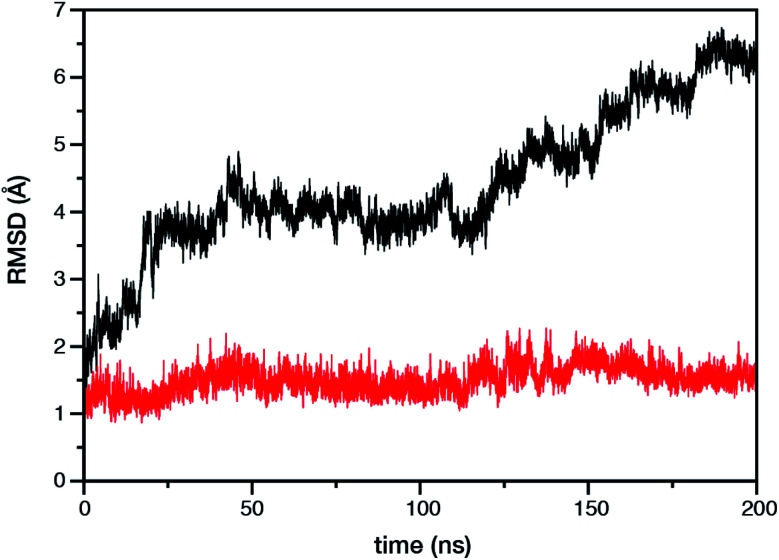
Time evolution of the root mean square deviation (RMSD) of the protein backbone atoms of the isolated CP47 protein. The black and red curves represent RMSD with and without the most flexible Ser480–Arg505 loop region, respectively. The reference structure in both cases is the CP47 protein configuration in “intact” PSII dimer.

**Fig. 6:**
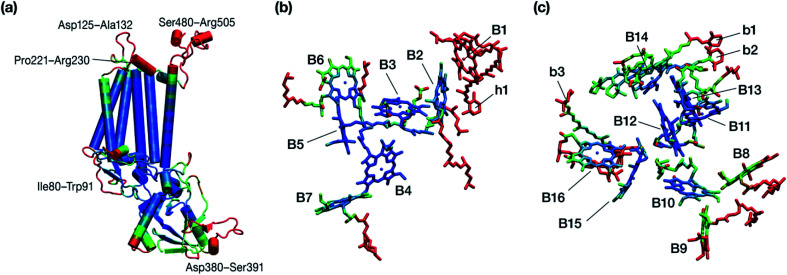
Graphical depiction of *B*-factors obtained from the production molecular dynamics simulations (200 ns) trajectory for the isolated CP47 protein (a) and its pigments (b and c). The blue and red colors represent the most rigid and most dynamic regions, respectively (not in scale; see Table 3 for explicit values). The *B*-factors are projected on structures taken from the initial state of the MD simulations that corresponds to the PSII-complexed, crystallographically consistent structure of CP47.

**Fig. 7:**
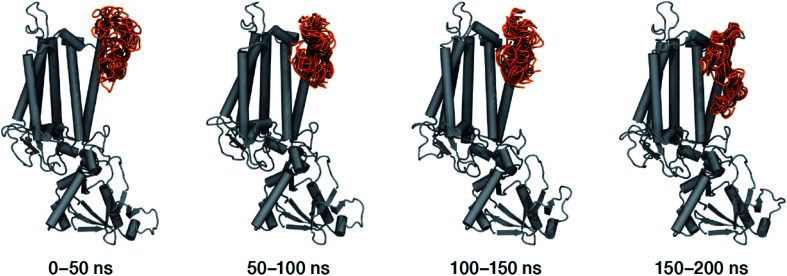
Conformational space spanned by the Ser480–Arg505 loop during the molecular dynamics simulations. The figures depict overlays of configurations adopted by this region (in orange) sampled over the four specified simulation intervals. This part of the CP47 polypeptide interacts over the stromal side of the membrane with the D2 and D1 proteins of assembled PSII, but in isolated CP47 it refolds entirely along one of the α-helices, over the side of the antenna that would normally form part of the interface with the D2 protein.

Within the time scale of our MD simulations we observe that the majority of the structured protein scaffold largely remains intact (Fig. 5 and 6). Especially the *trans*-membrane helices are the most stable region of the protein. However, we observe that the loop region extending from Ser480 to Arg505 is highly flexible and completely refolds in various conformations parallel to the transmembrane region during the course of the molecular dynamics (Fig. 7). In assembled PSII this part of CP47 interacts with both D2 and D1 proteins as it extends on top of them over the stromal side of the membrane. Already from the initial steps of our MD simulation the initial position is abandoned and the loop refolds along the transmembrane part of CP47 that is physiologically interfaced with the D2 chain. Fig. 7 depicts the progression of this refolding up to 200 ns of MD simulation. Based on a simple comparison with intact CP47 in the PSII dimer, it is obvious that such conformation of this loop region cannot possibly be adopted in intact PSII due to the presence of the adjacent core proteins. Therefore, isolation of the CP47 must lead inescapably to a drastic departure from the physiological conformation at least for this loop, which should affect considerably the pigments in its vicinity (both structurally and in terms of site energies), *i.e.* chlorophylls B8 and B9. This is also reflected in the *B*-factors for these two pigments, as shown in Table 3.

**Table 3:** Computed average mass-weighted *B*-factors (Å^2^) of the chlorophyll pigments from the molecular dynamics trajectories

Chlorophyll	Core ring atoms^a^	Chlorin ring^b^	Complete molecule^c^
B1	1333.48	1462.44	1395.05
B2	8.70	9.73	102.73
B3	4.50	5.08	30.97
B4	4.79	5.33	19.60
B5	4.58	5.08	14.11
B6	9.27	12.20	134.27
B7	9.70	11.34	85.11
B8	18.72	25.46	120.98
B9	20.58	29.48	170.63
B10	8.63	9.93	19.55
B11	7.15	8.16	20.16
B12	5.14	5.87	13.92
B13	5.27	5.99	27.11
B14	10.84	12.52	26.23
B15	6.79	7.75	229.20
B16	9.21	10.98	81.50

a Averaged over the central magnesium and the four coordinating nitrogen atoms.

b All atoms of the chlorin ring, without substituents.

c All atoms, *i.e.* the substituted macrocyclic ring and the complete phytyl chain.

Other parts of CP47 that are significantly destabilized within the time frame of the simulation are Asp125–Ala132, a loop that protrudes out of the membrane in the stromal side, but also the short helix Pro221–Arg230, an important region because of its proximity to PsbH in assembled PSII. Ile80–Trp91 is another highly flexible region that is normally found at the dimer interface and may interact with the PsbO chain of the *other* monomer in dimeric PSII. Finally, high flexibility is seen, as anticipated, in the lumenal part of the polypeptide, particularly the region Asp380–Ser391 (see Fig. 6), that does not bind chlorophylls but is spatially related to the B1 chlorophyll and the h1 carotenoid. This part of CP47 interacts with PsbU and D2, while it is also relatively close to the OEC region. It is anticipated that such effects are mutual, albeit to a still unknown extent. That is, the significant structural destabilization of this part of CP47 upon de-complexation from PSII implies that similar structural perturbations are to be expected in core complex preparations devoid of the intrinsic antenna proteins.

**Fig. 8:**
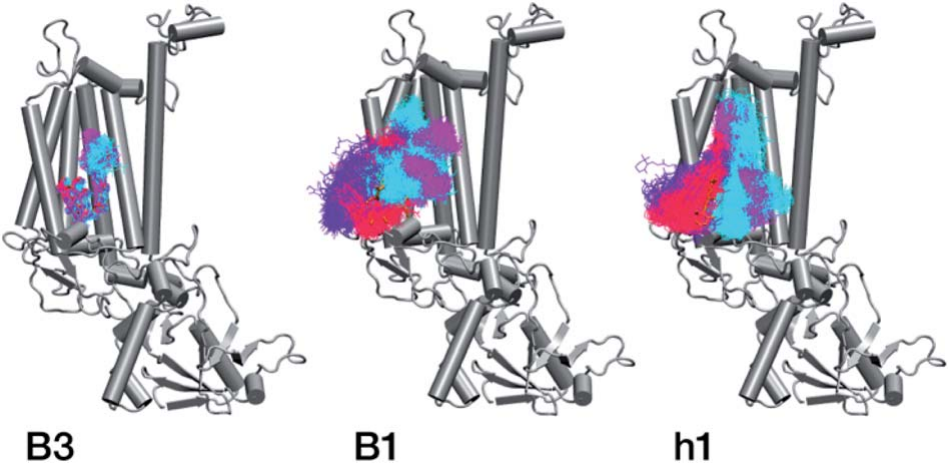
Conformational space spanned by chlorophylls B3 and B1 and carotenoid h1 during the MD simulations. Red, violet, cyan and magenta correspond to simulation intervals of 0–50 ns, 50–100 ns, 100–150 ns, and 150–200 ns, respectively. The simulations suggest that B1 and h1 cannot maintain their native positions in isolated CP47.

With respect to the embedded antenna pigments, we observed that the macrocyclic rings of most chlorophylls situated at the interior of the CP47 protein matrix remain stable. However, the results obtained for chlorophyll B1 show that it is extremely destabilized during the course of the simulation and adopts a very wide range of configurations (Fig. 6 and 8, Table 3), both in terms of fluxionality of the phytyl chain (whipping motion) and, most importantly, of the translational motion of the complete molecule. The results in Table 3 show a difference of two orders of magnitude in *B*-factor between B1 and the other chlorophylls, a numerical result that reflects the complete departure of the pigment from its original location. Overall, the conformational space of the B1 chromophore is very large and extends beyond the part of CP47 that would normally correspond to the transmembrane region, suggesting a high predisposition toward dissociation. The conformations seen in the MD simulations would have been impossible to adopt in the presence of the membrane and of other neighbouring proteins, particularly of PsbH. It may be surmised that, conversely, the presence of PsbH still complexed with isolated CP47 may help avoid the complete loss of B1. B1 is axially coordinated by a water molecule (see Table 1). Therefore, the departure of B1 from its initial position during the simulation is possible because it is not covalently linked to the protein matrix. However, this is not the *cause* of the observed dissociative trajectory, because chlorophylls B7 and B10, which also have water as axial ligand, are significantly more stable and display lower *B*-factors than many other covalently linked pigments (see Table 3).

The h1 β-carotene situated next to B1 was also highly mobile throughout the course of the simulation, in contrast to the other three β-carotenes that remained comparatively stable (Table 4, Fig. 8). Therefore, the MD simulations suggest that the B1 chlorophyll and the h1 β-carotene are the most conformationally dynamic pigments in isolated CP47. Due to their close proximity to B1 and h1, the geometric and electronic properties of chlorophylls B2, B8, and B9 are also likely to be significantly affected by the structural destabilization or loss of these pigments. This is consistent with the *B*-factors reported in Table 3.

**Table 4:** Computed average mass-weighted *B*-factors (Å^2^) of the β-carotene pigments from the molecular dynamics trajectories

Carotenoid	*B*-Factor
b1	53.55
b2	26.52
b3	64.43
h1	1114.18

The chlorophylls and the axial ligands in our MD simulations are connected through a harmonically restrained bond, which implies that chlorophylls bound axially to protein (His) ligands cannot be dissociated from the protein matrix. However, we also performed analogous MD simulations (for 200 ns) with no axial bond and found that all protein bound chlorophylls and the Mg–His distances remain stable throughout the simulation (see Fig. S2†), which confirms that all internal chlorophylls remain stable in the isolated CP47 complex, whereas B1 and h1 show similarly a highly dynamic behaviour.

In conclusion, the MD simulations show that isolated CP47 adopts non-native conformations of the protein and the pigments. As a result, the fitted or derived site energies based on experiments performed using isolated CP47 may not correlate well with the native system, or not to the degree that would allow safe conclusions regarding the latter. The results indicate that B1 and h1 may be lost upon extraction and isolation of CP47, or adopt conformations that have no relevance to the native system. In addition, isolation of the protein necessarily leads to the loss of key long-range electrostatics and inter-subunit interactions, while detergents typically employed in experimental studies could interfere with the protein and pigments, potentially inducing non-native conformational transitions and altering the local electrostatics around the pigment binding sites, thereby affecting their site energies. Therefore, the MD results obtained here question the integrity of protein–pigment interactions of isolated CP47 and the suitability of isolated CP47 as a safe platform for extrapolating to the native system.

## 4. Conclusions and perspectives

In this study we have studied the lowest excited states of the chlorophylls in the light-harvesting CP47 core antenna complex of dimeric cyanobacterial photosystem II using a QM/MM approach. We find that the order of site energies of the CP47 chromophores is modulated by the structured and anisotropic electrostatics of the protein matrix. The internally located chlorophyll B3 is computed to have the lowest site energy, a result that deviates from previous suggestions. This is followed by B1, which has been considered before as the pigment with the lowest site energy.^19^ By contrast, other popular hypotheses, including B9 and B16, are computed to be among the most blue-shifted chlorophylls. The site energies of B3 and B1 are of the same order in magnitude as the lowest excitonic states computed for the reaction centre,^74^ but the uncertainty in absolute values and the spread of values due to dynamics do not allow us to make concrete inferences regarding possible mechanisms of excitation energy transfer. The present results provide an alternative view of CP47 than those previously discussed in the literature, hopefully complementing other approaches to this fundamental problem of natural photosynthesis. A principal complicating factor in aligning and comparing different results is the nature of the sample used for experimental work or of the model used for computational studies. It remains impossible to reconcile all observations into a unique assignment, particularly since many of them are obtained from samples that should not be assumed to reflect the properties of dimeric cyanobacterial PSII as a matter of principle. Using molecular dynamics simulations we show that the isolated CP47 protein is not representative of the PSII bound intact CP47. Isolation triggers changes in the conformation of the protein, particularly in regions that physiologically interact with other core proteins of PSII. A large-scale structural change is documented for a specific part of the protein that interacts with the D1/D2 core dimer. Importantly, the MD simulations also suggest that the B1 chlorophyll and the h1 carotenoid are greatly destabilized and likely lost in isolated CP47. In total, the conformational changes evidenced by the present simulations give little confidence that studies on isolated CP47 can offer reliable insights into the physiological excitation profile of the antenna.

The above observations are transferable to other similar pigment–protein complexes. Extraction from their physiological state in assembled supercomplexes should be expected to alter the structure of the protein matrix and hence of the pigment binding sites, but also to reorganize the crucial electrostatic environment that largely defines the excitation profile of the embedded pigments. This calls for great caution when discussing experimental observations on isolated preparations: extrapolating to the natural system should be considered unjustifiable.

Our study models complexed cyanobacterial CP47, but the structure of this protein varies among species. In addition, CP47 may be directly interacting with specific external antenna proteins that are absent in the present PSII model. Such differences may have consequences for the identity of the red-most pigment, therefore the present work and the all-atom QM/MM model developed for the present purpose will be extended in the future to the study of different species and different PSII architectures. It would also be highly desirable to simulate the distribution of site energies for any given chromophore with explicit consideration of dynamics. This task is computationally intractable using the present high-level protocol for the quantum mechanics calculation of excitation energies, owing to the compound cost of the TD-DFT calculations themselves and the requirement for QM/MM optimization of all chromophores for any distinct protein conformation sampled during the MD simulation. Therefore, a lower-level but adequately reliable and benchmarked alternative that can operate directly on thousands of MD snapshots is needed. We are currently developing and testing semiempirical multiscale protocols that will be able to provide statistically meaningful approximated QM-quality site energies for protein-embedded biochromophores in general MD applications.

## Conflicts of interest

There are no conflicts to declare.

## Supplementary Material

SC-012-D0SC06616H-s001
