# Supplementary material for: Chlorophyll excitation energies and structural stability of the CP47 antenna of photosystem II: a case study in the first-principles simulation of light-harvesting complexes
Source: Chem Sci. 2021 Feb 12;12(12):4463–76. doi: 10.1039/d0sc06616h (PMC8179452; doi:10.1039/d0sc06616h)
Supplement: SC-012-D0SC06616H-s001 [file SC-012-D0SC06616H-s001.pdf]

Electronic Supplementary Material (ESI) for Chemical Science.
This journal is © The Royal Society of Chemistry 2021

Chlorophyll excitation energies and structural stability of the CP47 antenna of Photosystem II: a case study in the first-principles simulation of light-harvesting complexes

Abhishek Sirohiwal,[ab] Frank Neese,[a] Dimitrios A. Pantazis[a]*

[a] Max-Planck-Institut für Kohlenforschung, Kaiser-Wilhelm-Platz 1, 45470 Mülheim an der Ruhr, Germany

[b] Fakultät für Chemie und Biochemie, Ruhr-Universität Bochum, 44780 Bochum, Germany

Electronic Supplementary Information

Table S1. Computed site energies (in eV) of CP47 chromophores using the *ω*B97X-V functional with the Def2-TZVP basis set. Comparison of first excitation energies obtained *in vacuo*, in the electrostatic field of a PSII monomer, and in the electrostatic field of the PSII dimer. The pigment geometries in all cases are derived from QM/MM optimisations inside the complete PSII dimer. Parentheses indicate oscillator strengths.

**Site**	**Q_y (Vacuum)**	**Q_y (Monomer)**	**Q_y (Dimer)**
B1	1.941(0.22)	1.893 (0.24)	1.899 (0.25)
B2	1.928 (0.22)	1.948 (0.16)	1.953 (0.17)
B3	1.888 (0.23)	1.888 (0.19)	1.884 (0.19)
B4	1.935 (0.23)	1.928 (0.22)	1.947 (0.21)
B5	1.909 (0.22)	1.923 (0.17)	1.921 (0.17)
B6	1.924 (0.21)	1.923 (0.20)	1.933 (0.19)
B7	1.925 (0.23)	1.923 (0.19)	1.915 (0.21)
B8	1.951 (0.21)	1.998 (0.23)	1.967 (0.23)
B9	1.981 (0.22)	2.091 (0.20)	2.102 (0.20)
B10	1.965 (0.21)	1.974 (0.18)	1.966 (0.20)
B11	1.967 (0.23)	1.999 (0.24)	1.996 (0.24)
B12	1.939 (0.21)	1.944 (0.19)	1.941 (0.20)
B13	1.917 (0.23)	1.946 (0.25)	1.921 (0.27)
B14	1.942 (0.22)	1.957 (0.24)	1.956 (0.24)
B15	1.933 (0.22)	1.938 (0.20)	1.930 (0.21)
B16	1.945 (0.22)	2.009 (0.23)	2.050 (0.22)

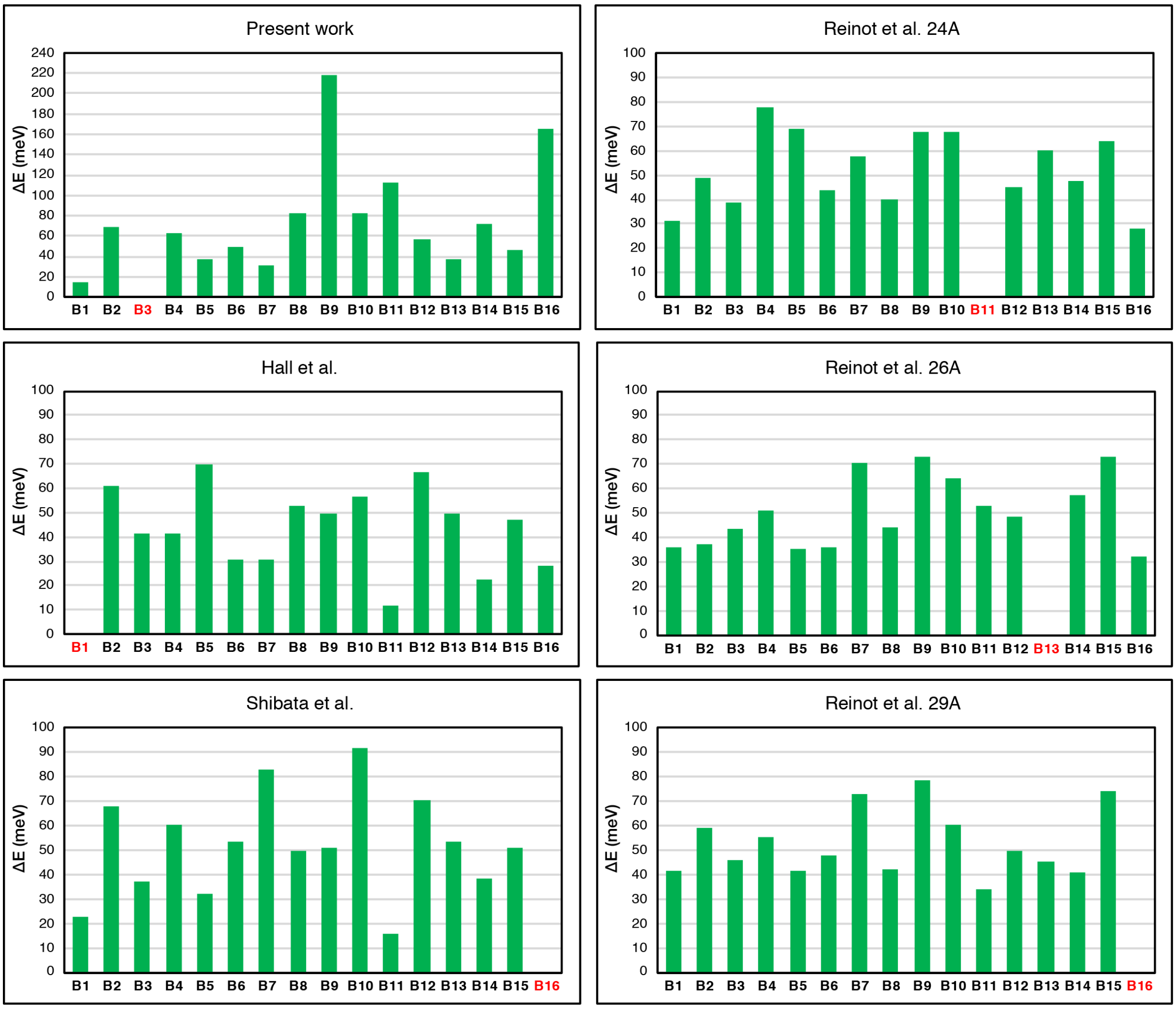

Figure S1. Comparison of vertical excitation energies for the CP47 chlorophylls computed in the present work with fitted site energies reported in selected past papers by Hall et al.,[1] Shibata et al.,[2] and Reinot et al.[3]

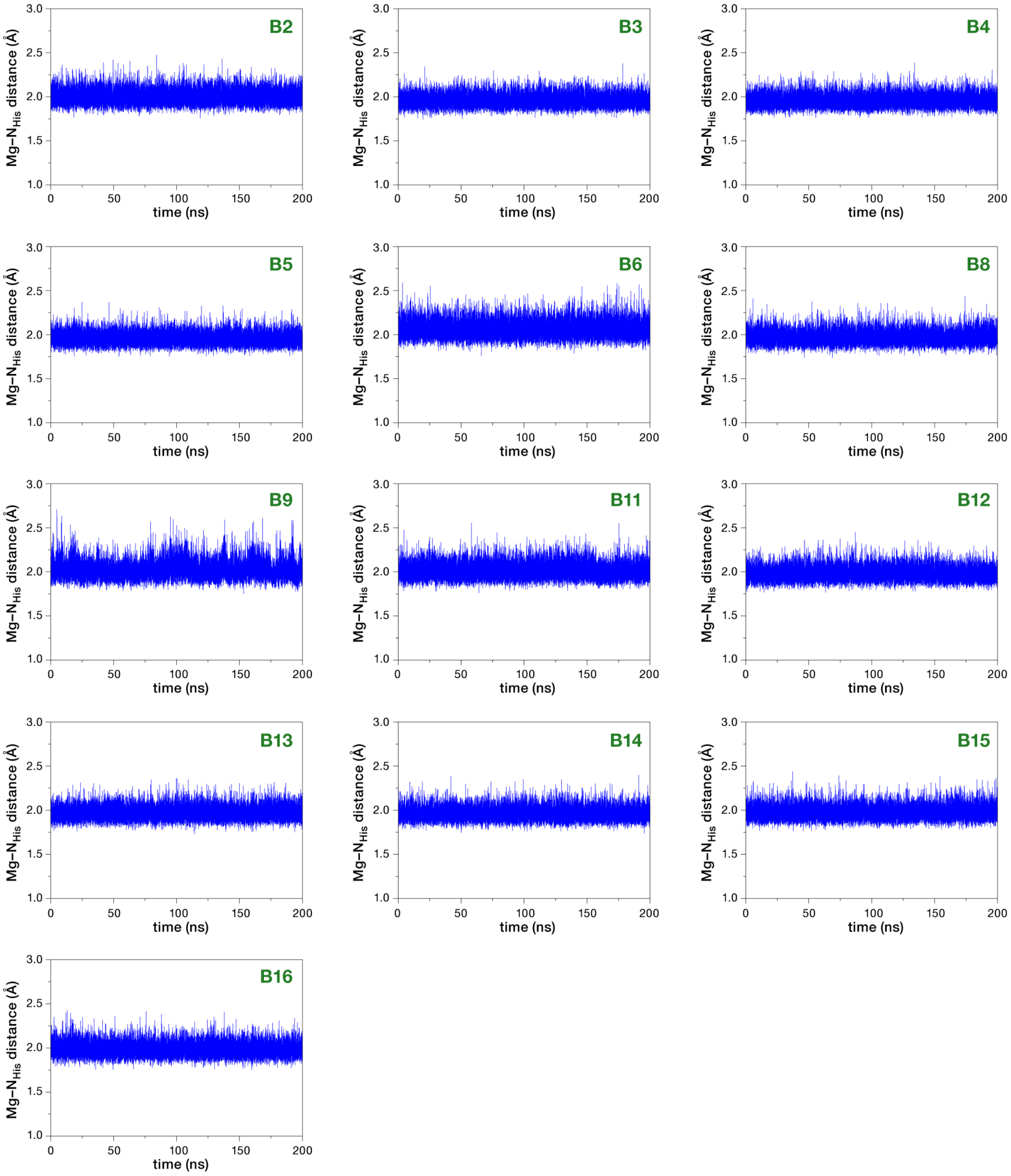

Figure S2. Distances between the Mg ion and the imidazole N atom for all CP47 chlorophylls that are formally coordinated by a histidine residue, from MD simulations carried out without specifying the presence of a Mg–N$_{His}$ bond.

COFACTOR FORCE FIELD PARAMETERS

Definition of atoms types, bond connectivity and RESP charges for Chlorophyll *a*

```
@<TRIPOS>MOLECULE
CLA
  137   145     1     0     0
SMALL
No Charge or Current Charge


@<TRIPOS>ATOM
      1 CAA        -7.0450    36.1740     7.9070 ct2     2740 CLA      -0.171036
      2 CAB        -3.7300    34.5260    -0.6930 cf      2740 CLA       0.004602
      3 CAC         2.5080    37.2040     3.4510 ct2     2740 CLA       0.051556
      4 CAD        -2.4990    36.0040     9.9420 c2k     2740 CLA       0.700109
      5 NA         -4.6920    34.9520     5.9050 ns      2740 CLA      -0.406343
      6 CBA        -6.8560    37.1890     6.7410 ct2     2740 CLA      -0.054130
      7 CBB        -4.6280    34.4210    -1.6550 c2      2740 CLA      -0.352117
      8 CBC         2.2640    38.7120     3.6060 ct3     2740 CLA      -0.161821
      9 CBD        -3.8660    35.5320     9.6270 ct1     2740 CLA      -0.684536
     10 NB         -3.7370    34.8980     3.0460 nmh     2740 CLA      -0.460890
     11 OBD        -2.0730    36.1940    11.1240 o2c     2740 CLA      -0.486229
     12 CGA        -7.7650    38.3670     6.9690 c2a     2740 CLA       0.661278
     13 CGD        -4.0530    34.1050    10.1490 c2a     2740 CLA       0.775843
     14 ND         -1.8910    35.7750     6.4000 nmh     2740 CLA      -0.562974
     15 CED        -4.3760    32.7210    12.2440 ct3     2740 CLA       0.023891
     16 CHA        -3.8660    35.4610     8.1530 csb     2740 CLA       0.215505
     17 CHB        -6.0140    34.3700     3.9770 cab     2740 CLA      -0.412162
     18 CHC        -1.8280    35.2750     1.4160 cab     2740 CLA      -0.294853
     19 CHD         0.3970    36.3220     5.6940 cab     2740 CLA      -0.409266
     20 CMA        -6.8010    32.6540     6.4090 ct3     2740 CLA      -0.284997
     21 CMB        -6.7410    33.8690     0.8460 ct3     2740 CLA      -0.414554
     22 CMC         0.9540    36.4980     0.5370 ct3     2740 CLA      -0.376255
     23 CMD         0.8770    36.7840     8.8420 ct3     2740 CLA      -0.347111
     24 C1         -8.6360    40.5350     6.0970 c3      2740 CLA       0.426615
     25 C1A        -4.8840    35.0930     7.1570 ccs     2740 CLA      -0.015834
     26 C1B        -5.0500    34.5490     2.8370 crb     2740 CLA       0.074017
     27 C1C        -0.8290    35.7730     2.3840 crb     2740 CLA       0.080581
     28 C1D        -0.6310    36.1660     6.7240 cpb     2740 CLA       0.331507
     29 O1A        -8.4760    38.5020     7.9790 o2c     2740 CLA      -0.524995
     30 O1D        -3.7340    33.1220     9.4970 o2c     2740 CLA      -0.495118
     31 C2         -8.5480    41.2380     4.7550 c2      2740 CLA      -0.554928
     32 C2A        -6.2910    34.8490     7.6040 ct1     2740 CLA       0.114777
     33 C2B        -5.4130    34.3160     1.4200 cbb     2740 CLA       0.215950
     34 C2C         0.4720    36.3340     1.9650 cbb     2740 CLA       0.152944
     35 C2D        -0.3890    36.3570     8.1300 cbb     2740 CLA       0.065292
     36 O2A        -7.7680    39.3980     5.9540 o1c     2740 CLA      -0.451704
     37 O2D        -4.1950    33.9620    11.5440 o1c     2740 CLA      -0.371917
     38 C3         -9.2210    40.8860     3.6530 c2      2740 CLA       0.294232
     39 C3A        -6.8630    34.2030     6.3470 ct1     2740 CLA       0.032573
     40 C3B        -4.1480    34.5460     0.7400 cbb     2740 CLA      -0.211021
     41 C3C         1.1310    36.5860     3.2470 cbb     2740 CLA      -0.231334
     42 C3D        -1.7150    36.0590     8.6940 cbb     2740 CLA      -0.247617
     43 C4        -10.1750    39.7320     3.6380 c3      2740 CLA      -0.407700
     44 C4A        -5.8190    34.5410     5.3130 ccs     2740 CLA       0.357959
     45 C4B        -3.1860    34.9180     1.8120 cnb     2740 CLA       0.260744
     46 C4C         0.1090    36.1780     4.2590 cnb     2740 CLA       0.314971
     47 C4D        -2.5370    35.7760     7.6260 cqb     2740 CLA       0.083937
     48 C5         -9.0260    41.6810     2.3920 c3      2740 CLA      -0.224157
     49 C6        -10.3080    42.4310     2.0720 c3      2740 CLA       0.110326
     50 C7        -10.2390    42.9460     0.6500 c3      2740 CLA      -0.030187
     51 C8        -11.5560    43.6590     0.3650 c3      2740 CLA       0.251557
     52 C9        -12.4020    42.7960    -0.5730 c3      2740 CLA      -0.368354
     53 C10       -11.2950    45.0990    -0.1070 c3      2740 CLA      -0.274480
     54 C11       -11.4660    46.1280     1.0390 c3      2740 CLA       0.184319
     55 C12       -10.1830    46.8020     1.5770 c3      2740 CLA      -0.230634
     56 C13       -10.2850    48.3140     1.8780 c3      2740 CLA       0.277050
     57 C14        -9.9740    49.1400     0.6290 c3      2740 CLA      -0.296828
     58 C15        -9.3560    48.7420     3.0260 c3      2740 CLA      -0.137174
     59 C16        -9.9310    49.9130     3.8350 c3      2740 CLA       0.075364
     60 C17        -9.5600    49.8850     5.3270 c3      2740 CLA      -0.187234
     61 C18        -9.7740    51.2180     6.0710 c3      2740 CLA       0.411365
     62 C19        -8.6700    51.5090     7.0880 c3      2740 CLA      -0.311376
     63 C20       -11.1490    51.3190     6.7470 c3      2740 CLA      -0.419125
     64 NC         -1.0080    35.7330     3.6970 ns      2740 CLA      -0.456670
     65 MG         -2.7780    34.9590     4.7430 mgc     2740 CLA       1.052289
     66 HAA1       -6.6740    36.6050     8.8370 hc      2740 CLA       0.070073
```

```
    67 HAA2         -8.1060    35.9710     8.0520 hc      2740 CLA       0.070073
    68 HAB          -2.6730    34.6010    -0.9480 ha      2740 CLA       0.090045
    69 HAC1          3.1610    36.9980     2.6020 hc      2740 CLA       0.027001
    70 HAC2          2.9950    36.7930     4.3350 hc      2740 CLA       0.027001
    71 HBA1         -7.0840    36.7140     5.7870 hc      2740 CLA       0.027329
    72 HBA2         -5.8180    37.5180     6.6920 hc      2740 CLA       0.027329
    73 HBB1         -5.6870    34.3460    -1.4060 ha      2740 CLA       0.146195
    74 HBB2         -4.3120    34.4070    -2.6970 ha      2740 CLA       0.146195
    75 HBC1          3.2160    39.2210     3.7560 hc      2740 CLA       0.050268
    76 HBC2          1.6170    38.8890     4.4650 hc      2740 CLA       0.050268
    77 HBC3          1.7850    39.0960     2.7050 hc      2740 CLA       0.050268
    78 HBD          -4.6400    36.1680    10.0570 hc      2740 CLA       0.191948
    79 HED1         -4.4560    32.9130    13.3140 h1      2740 CLA       0.067353
    80 HED2         -3.5210    32.0690    12.0570 h1      2740 CLA       0.067353
    81 HED3         -5.2860    32.2350    11.8940 h1      2740 CLA       0.067353
    82 HHB          -7.0180    34.0550     3.6920 ha      2740 CLA       0.149746
    83 HHC          -1.5410    35.1690     0.3700 ha      2740 CLA       0.171031
    84 HHD           1.4160    36.5550     6.0010 ha      2740 CLA       0.186781
    85 HMA1         -7.2180    32.2380     5.4930 hc      2740 CLA       0.076415
    86 HMA2         -7.3770    32.3020     7.2640 hc      2740 CLA       0.076415
    87 HMA3         -5.7640    32.3360     6.5130 hc      2740 CLA       0.076415
    88 HMB1         -6.6620    33.7960    -0.2390 hc      2740 CLA       0.125522
    89 HMB2         -7.5130    34.5930     1.1060 hc      2740 CLA       0.125522
    90 HMB3         -7.0040    32.8930     1.2560 hc      2740 CLA       0.125522
    91 HMC1          1.9520    36.9380     0.5390 hc      2740 CLA       0.117064
    92 HMC2          0.2710    37.1530    -0.0040 hc      2740 CLA       0.117064
    93 HMC3          0.9880    35.5260     0.0490 hc      2740 CLA       0.117064
    94 HMD1          0.6940    36.8250     9.9160 hc      2740 CLA       0.115985
    95 HMD2          1.1780    37.7700     8.4880 hc      2740 CLA       0.115985
    96 HMD3          1.6710    36.0660     8.6350 hc      2740 CLA       0.115985
    97 H2           -7.8830    42.0990     4.6830 ha      2740 CLA       0.197068
    98 H8          -12.1510    43.7760     1.2710 hc      2740 CLA      -0.021591
    99 H11          -8.3130    41.1840     6.9100 h1      2740 CLA      -0.003770
   100 H12          -9.6590    40.2300     6.3210 h1      2740 CLA      -0.003770
   101 H13         -11.3130    48.5030     2.1900 hc      2740 CLA      -0.037920
   102 H18          -9.7310    51.9800     5.2920 hc      2740 CLA      -0.061702
   103 H2A          -6.3720    34.2630     8.5200 hc      2740 CLA       0.008180
   104 H41         -10.6060    39.6290     2.6420 hc      2740 CLA       0.118056
   105 H42         -10.9710    39.9070     4.3620 hc      2740 CLA       0.118056
   106 H43          -9.6430    38.8170     3.8990 hc      2740 CLA       0.118056
   107 H3A          -7.8900    34.5280     6.1800 hc      2740 CLA       0.028468
   108 H51          -8.7610    41.0200     1.5670 hc      2740 CLA       0.065906
   109 H52          -8.2000    42.3840     2.5140 hc      2740 CLA       0.065906
   110 H61         -10.4410    43.2610     2.7670 hc      2740 CLA      -0.012985
   111 H62         -11.1680    41.7730     2.1930 hc      2740 CLA      -0.012985
   112 H71         -10.0860    42.1250    -0.0500 hc      2740 CLA       0.001818
   113 H72          -9.3980    43.6290     0.5270 hc      2740 CLA       0.001818
   114 H91         -13.3450    43.3000    -0.7790 hc      2740 CLA       0.081706
   115 H92         -12.6010    41.8340    -0.1000 hc      2740 CLA       0.081706
   116 H93         -11.8620    42.6370    -1.5060 hc      2740 CLA       0.081706
   117 H101        -11.9810    45.3450    -0.9190 hc      2740 CLA       0.063428
   118 H102        -10.2850    45.1720    -0.5100 hc      2740 CLA       0.063428
   119 H111        -11.9600    45.6280     1.8720 hc      2740 CLA      -0.028740
   120 H112        -12.1410    46.9120     0.6930 hc      2740 CLA      -0.028740
   121 H121         -9.3850    46.6500     0.8500 hc      2740 CLA       0.042399
   122 H122         -9.8840    46.2880     2.4910 hc      2740 CLA       0.042399
   123 H141        -10.0520    50.2010     0.8660 hc      2740 CLA       0.066213
   124 H142        -10.6860    48.8910    -0.1580 hc      2740 CLA       0.066213
   125 H143         -8.9630    48.9180     0.2880 hc      2740 CLA       0.066213
   126 H151         -8.3850    49.0260     2.6190 hc      2740 CLA       0.030146
   127 H152         -9.1870    47.8930     3.6900 hc      2740 CLA       0.030146
   128 H161        -11.0180    49.9090     3.7410 hc      2740 CLA      -0.011209
   129 H162         -9.5780    50.8490     3.4010 hc      2740 CLA      -0.011209
   130 H171         -8.5140    49.5960     5.4210 hc      2740 CLA       0.032589
   131 H172        -10.1500    49.1110     5.8190 hc      2740 CLA       0.032589
   132 H191         -8.8710    52.4590     7.5830 hc      2740 CLA       0.063258
   133 H192         -7.7080    51.5620     6.5770 hc      2740 CLA       0.063258
   134 H193         -8.6420    50.7120     7.8320 hc      2740 CLA       0.063258
   135 H201        -11.2360    52.2810     7.2530 hc      2740 CLA       0.092857
   136 H202        -11.2560    50.5160     7.4750 hc      2740 CLA       0.092857
   137 H203        -11.9310    51.2330     5.9940 hc      2740 CLA       0.092857
@<TRIPOS>BOND
     1     1     6 1
     2     1    32 1
     3     1    66 1
     4     1    67 1
     5     2     7 1
     6     2    40 1
     7     2    68 1
     8     3     8 1
```

9	3	41	1
10	3	69	1
11	3	70	1
12	4	9	1
13	4	11	1
14	4	42	1
15	5	25	1
16	5	44	1
17	5	65	1
18	6	12	1
19	6	71	1
20	6	72	1
21	7	73	1
22	7	74	1
23	8	75	1
24	8	76	1
25	8	77	1
26	9	13	1
27	9	16	1
28	9	78	1
29	10	26	1
30	10	45	1
31	10	65	1
32	12	29	1
33	12	36	1
34	13	30	1
35	13	37	1
36	14	28	1
37	14	47	1
38	14	65	1
39	15	37	1
40	15	79	1
41	15	80	1
42	15	81	1
43	16	25	1
44	16	47	1
45	17	26	1
46	17	44	1
47	17	82	1
48	18	27	1
49	18	45	1
50	18	83	1
51	19	28	1
52	19	46	1
53	19	84	1
54	20	39	1
55	20	85	1
56	20	86	1
57	20	87	1
58	21	33	1
59	21	88	1
60	21	89	1
61	21	90	1
62	22	34	1
63	22	91	1
64	22	92	1
65	22	93	1
66	23	35	1
67	23	94	1
68	23	95	1
69	23	96	1
70	24	31	1
71	24	36	1
72	24	99	1
73	24	100	1
74	25	32	1
75	26	33	1
76	27	34	1
77	27	64	1
78	28	35	1
79	31	38	1
80	31	97	1
81	32	39	1
82	32	103	1
83	33	40	1
84	34	41	1
85	35	42	1
86	38	43	1
87	38	48	1
88	39	44	1

```
    89    39   107 1
    90    40    45 1
    91    41    46 1
    92    42    47 1
    93    43   104 1
    94    43   105 1
    95    43   106 1
    96    46    64 1
    97    48    49 1
    98    48   108 1
    99    48   109 1
   100    49    50 1
   101    49   110 1
   102    49   111 1
   103    50    51 1
   104    50   112 1
   105    50   113 1
   106    51    52 1
   107    51    53 1
   108    51    98 1
   109    52   114 1
   110    52   115 1
   111    52   116 1
   112    53    54 1
   113    53   117 1
   114    53   118 1
   115    54    55 1
   116    54   119 1
   117    54   120 1
   118    55    56 1
   119    55   121 1
   120    55   122 1
   121    56    57 1
   122    56    58 1
   123    56   101 1
   124    57   123 1
   125    57   124 1
   126    57   125 1
   127    58    59 1
   128    58   126 1
   129    58   127 1
   130    59    60 1
   131    59   128 1
   132    59   129 1
   133    60    61 1
   134    60   130 1
   135    60   131 1
   136    61    62 1
   137    61    63 1
   138    61   102 1
   139    62   132 1
   140    62   133 1
   141    62   134 1
   142    63   135 1
   143    63   136 1
   144    63   137 1
   145    64    65 1
@<TRIPOS>SUBSTRUCTURE
     1 CLA         1 TEMP              0 ****  ****    0 ROOT
```

Bonded and LJ parameters for Chlorophyll *a*

```
Force Field modification file for CLA
MASS
mgc 24.305        0.120              magnesium
csb 12.01         0.360              Inner Sp2 carbons in conjugated systems
cab 12.01         0.360              Inner Sp2 carbons in conjugated systems
ccs 12.01         0.360              Inner Sp2 carbons in conjugated systems
ct1 12.01         0.878              Sp3 C
ct2 12.01         0.878              Sp3 C
ct3 12.01         0.878              Sp3 C
crb 12.01         0.360              Sp2 carbons in non-pure aromatic systems
cbb 12.01         0.360              Sp2 carbons in non-pure aromatic systems
cnb 12.01         0.360              Sp2 carbons in non-pure aromatic systems
cpb 12.01         0.360              Sp2 carbons in non-pure aromatic systems
cqb 12.01         0.360              Sp2 carbons in non-pure aromatic systems
c2k 12.01         0.616              Sp2 C carbonyl group
```

```
c2a 12.01         0.616               Sp2 C carbonyl group
nmh 14.01         0.530               Inner Sp2 N in conjugated systems
ns  14.01         0.530               Inner Sp2 N in conjugated systems
o2c 16.00         0.434               Oxygen with one connected atom
o1c 16.00         0.465               Ether and ester oxygen

BOND
nmh   mgc    50.00   2.05
ns    mgc    50.00   2.150
nmh   cnb   316.00   1.375
nmh   crb   316.00   1.380
ns    cnb   316.00   1.375
ns    crb   316.00   1.380
nmh   cpb   316.00   1.393
nmh   cqb   316.00   1.348
cnb   cbb   263.00   1.456
crb   cbb   263.00   1.439
cpb   cbb   263.00   1.441
cqb   cbb   283.00   1.421
cbb   cbb   340.00   1.402
cbb   ct2   323.50   1.513               # Same with c3-ca
cbb   ct3   200.00   1.497
cbb   c2e   190.00   1.479
ct3   c2e   170.00   1.522
ns    ccs   296.00   1.362
ccs   ct1   250.00   1.527
ct1   ct1   250.00   1.552
ct1   ct2   250.00   1.541
ct1   ct3   250.00   1.541
ct1   hc    358.00   1.090
c2k   o2c   700.00   1.226
c2k   cbb   263.00   1.455
c2k   ct1   170.00   1.604
csb   ct1   250.00   1.525
cqb   csb   305.00   1.404
cnb   cab   350.00   1.404
crb   cab   350.00   1.406
cpb   cab   350.00   1.407
ccs   cab   370.00   1.380
ccs   csb   370.00   1.370
cab   ha    376.00   1.092
ct3   hc    358.00   1.090
c2a   o2c   700.00   1.222
c2a   o1c   380.00   1.363
o1c   ct2   358.00   1.457
o1c   ct3   358.00   1.457
o1c   c3    358.00   1.457
c2a   ct1   250.00   1.522
c2a   ct2   250.00   1.522
ct2   ct2   260.00   1.526
ct2   ct3   260.00   1.526
ct2   hc    358.00   1.090
ct3   hc    358.00   1.090
ct3   h1    358.00   1.090
c2e   o2c   650.00   1.229
cbb   cf    562.40   1.338 # same as ce - cf.

ANGLE
nmh   mgc   ns        50.0     87.0
nmh   mgc   nmh       50.0    158.39
ns    mgc   ns        50.0    158.06
mgc   nmh   cnb       70.0    125.45
mgc   nmh   cpb       70.0    130.82
mgc   nmh   cqb       70.0    122.4
mgc   nmh   crb       70.0    126.83
mgc   ns    cnb       70.0    128.15   # Same with mgc-nmh-cnb
mgc   ns    crb       70.0    126.4   # Same with mgc-nmh-crb
mgc   ns    ccs       70.0    125.8
cnb   nmh   crb      100.0    107.18
cnb   ns    crb      100.0    106.62   # Same with cnb-nmh-crb
cpb   nmh   cqb      100.0    106.17
nmh   cnb   cbb       70.0    109.5
nmh   crb   cbb       70.0    110.28
ns    cnb   cbb       70.0    109.72  # Same with nmh-cnb-cbb
ns    crb   cbb       70.0    110.28   # Same with nmh-crb-cbb
nmh   cpb   cbb       70.0    109.75
nmh   cqb   cbb       70.0    112.23
cnb   cbb   cbb       63.0    106.19
crb   cbb   cbb       63.0    106.41
cpb   cbb   cbb       63.0    104.92
```

```
cqb   cbb   cbb       63.0    106.92
nmh   cnb   cab       70.0    124.65
nmh   crb   cab       70.0    124.5
ns    cnb   cab       70.0    123.87   # Same with nmh-cnb-cab
ns    crb   cab       70.0    124.78   # Same with nmh-crb-cab
nmh   cpb   cab       70.0    122.04
nmh   cqb   csb       70.0    133.38
cbb   cbb   c2k       63.0    142.98
cbb   cbb   ct2       63.0    129.0
cbb   cbb   ct3       63.0    129.0
crb   cbb   ct3       63.0    123.5
cpb   cbb   ct3       63.0    127.11
cnb   cbb   c2e       63.0    124.4
cnb   cbb   ct2       63.0    124.4              # Same with cnb-cbb-c2e
cbb   cbb   c2e       63.0    128.5
cbb   cnb   cab       63.0    126.26
cbb   crb   cab       63.0    126.5
cbb   cpb   cab       63.0    128.14
ccs   ns    ccs      120.0    109.95
ns    ccs   ct1       70.0    110.59
ccs   ct1   ct1       63.0    101.61
ns    ccs   cab       70.0    125.5
ns    ccs   csb       70.0    123.21
ccs   ct1   ct2       63.0    112.1
ccs   ct1   ct3       63.0    112.1
ct1   ct1   ct2       63.0    114.0
ct1   ct1   ct3       63.0    114.0
ct1   ct2   ct2       63.0    114.0
ct1   ct2   ct3       63.0    114.0
cbb   ct2   ct3       63.250  112.090           # Same with c3-c3-ca
ccs   ct1   hc        35.0    109.5
ct1   ct1   hc        35.0    109.5
ct1   ct2   hc        35.0    109.5
cbb   ct2   hc        46.960  110.15            # Same with ca-c3-hc
ct2   ct1   hc        35.0    109.5
cbb   c2k  ct1        63.0    105.16
cbb   c2k  o2c        80.0    130.7
ct1   c2k  o2c        80.0    124.0
c2k   ct1  csb        63.0    104.67
cqb   csb  ct1        63.0    106.69
cqb   cbb  c2k        63.0    109.41
ct1   csb  ccs        63.0    128.26
c2k   ct1  c2a        63.0    111.0
csb   ct1  c2a        63.0    112.5
csb   ct1  hc         35.0    109.5
c2k   ct1  hc         35.0    109.5
csb   cqb  cbb        63.0    114.03
cnb   cab  cpb        63.0    126.9             # Same with csb-cqb-ccs
cnb   cab   ccs       63.0    129.9
crb   cab   ccs       63.0    127.92
crb   cab   cnb       63.0    128.48
cqb   csb   ccs       63.0    123.92
cnb   cab   ha        25.0    116.33
crb   cab   ha        25.0    115.48
cpb   cab   ha        25.0    116.72
ccs   cab   ha        25.0    115.98
cab   ccs   ct1       63.0    122.0
csb   ccs   ct1       63.0    125.8
c2a   o1c  ct2        45.0    106.0
c2a   o1c  ct3        45.0    106.0
c2a   o1c  c3         45.0    106.0
o1c   c2a  ct1        70.0    111.5
o1c   c2a  ct2        70.0    111.5
o2c   c2a  ct1        90.0    126.5
o2c   c2a  ct2        90.0    126.5
o2c   c2a  o1c       100.0    122.0
c2a   ct1  hc         35.0    109.5
c2a   ct2  hc         35.0    109.5
o1c   ct2  hc         35.0    109.5
o1c   ct2  h1         35.0    109.5
o1c   ct3  hc         35.0    109.5
o1c   ct3  h1         35.0    109.5
o1c   c3   h1         35.0    109.5
c2a   ct2  ct2        63.0    116.6
cbb   ct3   hc        35.0    109.5
cbb   c2e   o2c       80.0    121.9
cbb   c2e   ct3       63.0    118.0
ct1   ct3   ct3       40.0    114.2
ct1   ct3   hc        35.0    109.5
ct3   ct1   hc        35.0    109.5
```

```
ct3   c2e   o2c       80.0    122.4
c2e   ct3   hc        35.0    109.5
ct2   ct2   hc        35.0    109.5
ct2   ct3   hc        35.0    109.5
ct3   ct2   hc        35.0    109.5
ct3   ct3   hc        35.0    109.5
hc    ct2   hc        35.0    109.5
hc    ct3   hc        35.0    109.5
h1    ct3   h1        35.0    109.5
o2c   cqo   cq2       60.0    120.0
o1c   cq2   cqo       60.0    120.0
o1c   cq2   cq2       60.0    120.0
cq2   o1c   ct3       60.0    112.0
cqo   cq2   cq2       30.0    120.0
cq2   cqo   cq2       30.0    120.0
cqo   cq2   ct2       50.0    115.7
cq2   cq2   ct2       50.0    124.1
cqo   cq2   ct3       50.0    115.2
cq2   cq2   ct3       50.0    124.9
cq2   ct2   cqq       50.0    112.0
ct2   ct2   cqq       50.0    112.0
o1c   ct2   cqq       50.0    109.6
cq2   cqq   ct2       50.0    125.9
cqq   cq2   ct2       50.0    120.2
cqq   cq2   ct3       50.0    123.7
ct2   cq2   ct3       50.0    116.0
ct3   cq2   ct3       50.0    116.0
cq2   ct2   ct2       50.0    114.5
cq2   ct2   ct3       50.0    114.5
hc    ct2   cq2       35.0    109.5
hc    ct3   cq2       35.0    109.5
hc    ct2   cqq       35.0    109.5
on    no    ct2       80.0    109.5
on    no    ct3       80.0    109.5
ct3   no    ct2       60.0    109.5
ct3   no    ct3       60.0    109.5
no    ct2   ct2       67.7    115.0
no    ct2   hc        40.0    109.5
no    ct3   hc        40.0    109.5
ct2   ct2   ct2       58.0    109.5
ct2   ct2   ct3       58.0    109.5
ct2   ct1   ct3       58.0    111.5
ct2   ct1   ct2       58.0    111.5
ct3   ct1   ct2       58.0    111.5
ct3   ct1   ct3       58.0    111.5
o1c   c3    c2        50.0    109.6 # Same as o1c - ct2 - cqq
cbb   cbb   cf        65.490  124.050 # Same as      ce - ce - cf
cnb   cbb   cf        65.490  124.050 # Same as      ce - ce - cf
cbb   cf    c2        65.420  124.330 # Same as ce - cf - cf
cbb   cf    ha        50.240  117.980 # Same as ce - cf - ha

DIHE
X    nmh  mgc  X   1    0.000       180.000           2.000
X    ns   mgc  X   1    0.000       180.000           2.000
X    nmh  cnb  X   1    2.500       180.000           2.000
X    nmh  crb  X   1    2.500       180.000           2.000
X    ns   ccs  X   1    2.500       180.000           2.000
X    ns   cnb  X   1    2.500       180.000           2.000  # Same with X-nmh-cnb-X
X    ns   crb  X   1    2.500       180.000           2.000  # Same with X-nmh-crb-X
X    nmh  cpb  X   1    0.000       180.000           2.000
X    nmh  cqb  X   1    2.500       180.000           2.000
X    cnb  cbb  X   1    3.50        180.000           2.000
X    crb  cbb  X   1    3.50        180.000           2.000
X    cpb  cbb  X   1    3.50        180.000           2.000
X    cqb  cbb  X   1    3.50        180.000           2.000
X    cbb  cbb  X   1    5.00        180.000           2.000
ct1  ct1  ccs  X   1    1.575       180.000           2.000
hc   ct1  ccs  X   1    0.00        180.000           2.000
ct2  ct1  ccs  X   1    0.00        180.000           2.000
ct3  ct1  ccs  X   1    0.00        180.000           2.000
X    ct1  ct1  X   1    0.00        180.000           3.000
X    cbb  c2k  X   1    5.000       180.000           2.000
csb  ct1  c2k  X   1    1.575       180.000           2.000
hc   ct1  c2k  X   1    0.000       180.000           2.000
c2a  ct1  c2k  X   1    0.000       180.000           2.000
c2k  ct1  csb  X   1    1.575       180.000           2.000
hc   ct1  csb  X   1    0.000       180.000           2.000
c2a  ct1  csb  X   1    0.000       180.000           2.000
X    cab  ccs  X   1    3.500       180.000           2.000
X    csb  ccs  X   1    3.500       180.000           2.000
```

```
X    cab  cnb  X    1    3.500       180.000            2.000
X    cab  crb  X    1    3.500       180.000            2.000
X    cab  cpb  X    1    3.500       180.000            2.000
X    csb  cqb  X    1    3.500       180.000            2.000
o2c  c2a  o1c  X    1    1.000       180.000            1.000
o2c  c2a  o1c  c3   1    1.000       180.000            1.000
o2c  c2a  o1c  ct3  1    1.000       180.000            1.000
o2c  c2a  o1c  c3   1    6.000       180.000            2.000
o2c  c2a  o1c  X    1    6.000       180.000            2.000
ct1  c2a  o1c  X    1    1.500       180.000            2.000
ct1  c2a  o1c  ct3  1    1.500       180.000            2.000
ct2  c2a  o1c  X    1    1.500       180.000            2.000
ct2  c2a  o1c  c3   1    1.500       180.000            2.000
o1c  c2a  ct2  X    1    0.030         0.000            3.000
o1c  c2a  ct2  hc   1    0.030         0.000            3.000
o1c  c2a  ct2  ct2  1    0.030         0.000            3.000
o2c  c2a  ct2  hc   1    0.030       180.000            3.000
o2c  c2a  ct2  ct2  1    0.030       180.000            3.000
X    c2a  ct1  X    1    0.000       180.000            3.000
X    o1c  ct2  X    1    0.180         0.000            3.000
c2a  o1c  c3   c2   1    0.180         0.000            3.000
X    o1c  ct3  hc   1    0.180         0.000            3.000
c2a  o1c  c3   h1   1    0.180         0.000            3.000
c2a  o1c  ct3  h1   1    0.180         0.000            3.000
X    cbb  c2e  X    1    5.000       180.000            2.000
X    cbb  ct3  X    1    0.000       180.000            2.000
X    ct1  ct2  X    1    0.156         0.000            3.000
X    ct1  ct3  X    1    0.156         0.000            3.000
X    ct2  ct2  X    1    0.156         0.000            3.000
X    ct2  ct3  X    1    0.156         0.000            3.000
X    ct3  ct3  X    1    0.156         0.000            3.000
X    c2e  ct3  X    1    0.000       180.000            2.000
X    cbb  cf   X    4   26.600       180.000            2.000 # Same with X-ce-cf-X
X    cbb  ct2  X    2    0.000       180.000            2.000 # Same with X-c3-ca-X

IMPROPER
X X csb X        1.100 180.0    2.0
X X ns  X        1.100 180.0    2.0
X X nmh X        1.100 180.0    2.0
X X cab X        1.100 180.0    2.0
X X cbb X        1.100 180.0    2.0
X X ccs X        1.100 180.0    2.0
X X cnb X        1.100 180.0    2.0
X X crb X        1.100 180.0    2.0
X X ccs X        1.100 180.0    2.0
X X cpb X        1.100 180.0    2.0
X X cqb X        1.100 180.0    2.0
X X c2k o2c     10.500 180.0    2.0

NONBON
mgc     0.650   0.250
cab     1.908   0.086
csb     1.908   0.086
cbb     1.908   0.086
ccs     1.908   0.086
cnb     1.908   0.086
cpb     1.908   0.086
cqb     1.908   0.086
crb     1.908   0.086
c2k     1.908   0.086
c2a     1.908   0.086
o1c     1.6637  0.170
o2c     1.6612  0.210
nmh     1.824   0.170
ns      1.824   0.170
ct1     1.908   0.1094
ct2     1.908   0.1094
ct3     1.908   0.1094
```

Definition of the GAFF2 based atoms types, bond connectivity and RESP charges for β-carotene

```
@<TRIPOS>MOLECULE
BCR
   96    97     1     0     0
SMALL
No Charge or Current Charge


@<TRIPOS>ATOM
      1 C1           5.2430    34.2110     7.1440 c3      2741 BCR       0.520876
      2 C2           4.3100    34.8670     8.1640 c3      2741 BCR      -0.153725
      3 C3           4.2420    34.3760     9.5990 c3      2741 BCR       0.057544
      4 C4           4.2390    32.8230     9.7110 c3      2741 BCR      -0.129668
      5 C5           4.8880    32.1110     8.5550 c2      2741 BCR       0.189555
      6 C6           5.3500    32.7170     7.3970 ce      2741 BCR      -0.406682
      7 C7           5.9500    31.8730     6.3290 ce      2741 BCR       0.118700
      8 C8           7.0420    31.0820     6.5130 cf      2741 BCR      -0.350037
      9 C9           7.4690    30.2260     5.4030 cf      2741 BCR       0.188330
     10 C10          8.4190    29.3240     5.6480 ce      2741 BCR      -0.316233
     11 C11          8.8860    28.3120     4.6990 ce      2741 BCR      -0.008810
     12 C12          9.7950    27.4720     5.2180 cf      2741 BCR      -0.376308
     13 C13         10.3210    26.2840     4.4570 cf      2741 BCR       0.221509
     14 C14         11.1310    25.4070     5.1140 ce      2741 BCR      -0.341417
     15 C15         11.6470    24.1590     4.5150 ce      2741 BCR      -0.112451
     16 C16         12.3400    23.2780     5.2460 cf      2741 BCR      -0.126191
     17 C17         12.7450    21.9790     4.6920 cf      2741 BCR      -0.332899
     18 C18         13.2860    20.9250     5.3860 ce      2741 BCR       0.206356
     19 C19         13.5180    19.6680     4.6210 ce      2741 BCR      -0.365331
     20 C20         14.1140    18.5980     5.1740 cf      2741 BCR      -0.007725
     21 C21         14.1690    17.3900     4.3260 cf      2741 BCR      -0.336895
     22 C22         14.7030    16.1910     4.6980 ce      2741 BCR       0.170995
     23 C23         14.5980    15.0460     3.7480 ce      2741 BCR      -0.315312
     24 C24         14.6160    13.7720     4.2080 cf      2741 BCR       0.054632
     25 C25         14.3950    12.5470     3.3440 cf      2741 BCR      -0.300273
     26 C26         13.1670    12.3560     2.8240 c2      2741 BCR       0.178004
     27 C27         12.8460    11.1670     1.9330 c3      2741 BCR      -0.100874
     28 C28         14.0880    10.8480     1.1190 c3      2741 BCR       0.041870
     29 C29         15.0930    10.3820     2.1360 c3      2741 BCR      -0.174348
     30 C30         15.4560    11.5200     3.0820 c3      2741 BCR       0.517469
     31 C31          6.6780    34.7870     7.0350 c3      2741 BCR      -0.417696
     32 C32          4.5400    34.5790     5.8530 c3      2741 BCR      -0.456532
     33 C33          4.9680    30.5960     8.7790 c3      2741 BCR      -0.379835
     34 C34          6.8390    30.3420     4.0290 c3      2741 BCR      -0.258093
     35 C35          9.9310    26.0930     3.0060 c3      2741 BCR      -0.204429
     36 C36         13.6120    20.9330     6.8650 c3      2741 BCR      -0.168116
     37 C37         15.3170    16.0070     6.0540 c3      2741 BCR      -0.235548
     38 C38         12.0740    13.3510     3.0770 c3      2741 BCR      -0.415232
     39 C39         16.6330    12.2520     2.4630 c3      2741 BCR      -0.454504
     40 C40         15.6970    10.9170     4.4460 c3      2741 BCR      -0.431307
     41 HC21         3.3000    34.8060     7.7580 hc      2741 BCR       0.033746
     42 HC22         4.5730    35.9250     8.2030 hc      2741 BCR       0.033746
     43 HC31         3.3410    34.7700    10.0700 hc      2741 BCR      -0.005016
     44 HC32         5.0910    34.7730    10.1540 hc      2741 BCR      -0.005016
     45 HC41         3.2090    32.4800     9.8000 hc      2741 BCR       0.049649
     46 HC42         4.7510    32.5380    10.6300 hc      2741 BCR       0.049649
     47 HC7          5.4890    31.8890     5.3410 ha      2741 BCR       0.089803
     48 HC8          7.5830    31.0840     7.4600 ha      2741 BCR       0.196276
     49 H10C         8.8850    29.3400     6.6330 ha      2741 BCR       0.158878
     50 H11C         8.5300    28.2450     3.6710 ha      2741 BCR       0.163465
     51 H12C        10.1660    27.6560     6.2270 ha      2741 BCR       0.206724
     52 H14C        11.4150    25.6400     6.1410 ha      2741 BCR       0.159668
     53 H15C        11.4610    23.9520     3.4610 ha      2741 BCR       0.182949
     54 H16C        12.6090    23.5290     6.2720 ha      2741 BCR       0.182269
     55 H17C        12.6010    21.8380     3.6210 ha      2741 BCR       0.155445
     56 H19C        13.1950    19.6140     3.5820 ha      2741 BCR       0.202424
     57 H20C        14.5290    18.6200     6.1810 ha      2741 BCR       0.160907
     58 H21C        13.7500    17.4630     3.3230 ha      2741 BCR       0.170255
     59 H23C        14.5070    15.2350     2.6790 ha      2741 BCR       0.181715
     60 H24C        14.8020    13.6180     5.2710 ha      2741 BCR       0.106958
     61 H271        12.0080    11.3970     1.2760 hc      2741 BCR       0.037749
     62 H272        12.5530    10.3080     2.5350 hc      2741 BCR       0.037749
     63 H281        14.4460    11.7250     0.5800 hc      2741 BCR       0.000153
     64 H282        13.8880    10.0760     0.3760 hc      2741 BCR       0.000153
     65 H291        15.9900    10.0210     1.6310 hc      2741 BCR       0.040575
     66 H292        14.6860     9.5450     2.7020 hc      2741 BCR       0.040575
     67 H311         7.2350    34.2350     6.2790 hc      2741 BCR       0.094143
     68 H312         7.1800    34.6930     7.9980 hc      2741 BCR       0.094143
```

```
     69 H313         6.6270    35.8390     6.7540 hc      2741 BCR       0.094143
     70 H321         5.0960    34.1720     5.0080 hc      2741 BCR       0.102362
     71 H322         4.4860    35.6630     5.7650 hc      2741 BCR       0.102362
     72 H323         3.5330    34.1630     5.8570 hc      2741 BCR       0.102362
     73 H331         5.4430    30.1270     7.9180 hc      2741 BCR       0.101612
     74 H332         3.9630    30.1950     8.9040 hc      2741 BCR       0.101612
     75 H333         5.5550    30.3920     9.6740 hc      2741 BCR       0.101612
     76 H341         7.2990    29.6190     3.3560 hc      2741 BCR       0.076108
     77 H342         6.9930    31.3490     3.6410 hc      2741 BCR       0.076108
     78 H343         5.7690    30.1410     4.1010 hc      2741 BCR       0.076108
     79 H351        10.4000    25.1890     2.6200 hc      2741 BCR       0.062642
     80 H352        10.2640    26.9520     2.4240 hc      2741 BCR       0.062642
     81 H353         8.8470    26.0010     2.9290 hc      2741 BCR       0.062642
     82 H361        14.0300    19.9680     7.1510 hc      2741 BCR       0.054733
     83 H362        12.7030    21.1180     7.4370 hc      2741 BCR       0.054733
     84 H363        14.3380    21.7190     7.0750 hc      2741 BCR       0.054733
     85 H371        15.6820    14.9840     6.1520 hc      2741 BCR       0.071622
     86 H372        14.5670    16.1990     6.8210 hc      2741 BCR       0.071622
     87 H373        16.1470    16.7030     6.1760 hc      2741 BCR       0.071622
     88 H381        11.1620    13.0250     2.5780 hc      2741 BCR       0.105731
     89 H382        12.3730    14.3250     2.6890 hc      2741 BCR       0.105731
     90 H383        11.8940    13.4290     4.1490 hc      2741 BCR       0.105731
     91 H391        17.4330    11.5420     2.2530 hc      2741 BCR       0.100119
     92 H392        16.9940    13.0120     3.1560 hc      2741 BCR       0.100119
     93 H393        16.3180    12.7270     1.5350 hc      2741 BCR       0.100119
     94 H401        16.4630    10.1450     4.3720 hc      2741 BCR       0.090657
     95 H402        14.7720    10.4750     4.8180 hc      2741 BCR       0.090657
     96 H403        16.0300    11.6940     5.1340 hc      2741 BCR       0.090657
@<TRIPOS>BOND
     1     1     2 1
     2     1     6 1
     3     1    31 1
     4     1    32 1
     5     2     3 1
     6     2    41 1
     7     2    42 1
     8     3     4 1
     9     3    43 1
    10     3    44 1
    11     4     5 1
    12     4    45 1
    13     4    46 1
    14     5     6 2
    15     5    33 1
    16     6     7 1
    17     7     8 2
    18     7    47 1
    19     8     9 1
    20     8    48 1
    21     9    10 2
    22     9    34 1
    23    10    11 1
    24    10    49 1
    25    11    12 2
    26    11    50 1
    27    12    13 1
    28    12    51 1
    29    13    14 2
    30    13    35 1
    31    14    15 1
    32    14    52 1
    33    15    16 2
    34    15    53 1
    35    16    17 1
    36    16    54 1
    37    17    18 2
    38    17    55 1
    39    18    19 1
    40    18    36 1
    41    19    20 2
    42    19    56 1
    43    20    21 1
    44    20    57 1
    45    21    22 2
    46    21    58 1
    47    22    23 1
    48    22    37 1
    49    23    24 2
    50    23    59 1
    51    24    25 1
```

```
    52    24    60 1
    53    25    26 2
    54    25    30 1
    55    26    27 1
    56    26    38 1
    57    27    28 1
    58    27    61 1
    59    27    62 1
    60    28    29 1
    61    28    63 1
    62    28    64 1
    63    29    30 1
    64    29    65 1
    65    29    66 1
    66    30    39 1
    67    30    40 1
    68    31    67 1
    69    31    68 1
    70    31    69 1
    71    32    70 1
    72    32    71 1
    73    32    72 1
    74    33    73 1
    75    33    74 1
    76    33    75 1
    77    34    76 1
    78    34    77 1
    79    34    78 1
    80    35    79 1
    81    35    80 1
    82    35    81 1
    83    36    82 1
    84    36    83 1
    85    36    84 1
    86    37    85 1
    87    37    86 1
    88    37    87 1
    89    38    88 1
    90    38    89 1
    91    38    90 1
    92    39    91 1
    93    39    92 1
    94    39    93 1
    95    40    94 1
    96    40    95 1
    97    40    96 1
@<TRIPOS>SUBSTRUCTURE
     1 BCR         1 TEMP              0 ****  ****    0 ROOT
```

GAFF2 based bonded and LJ parameters for β-carotene

```
Remark line goes here
MASS

BOND #Associated parameters for the bonded atoms can be found in the AMBER GAFF2 force-field
library.

ANGLE #Associated parameters for the angles can be found in the AMBER GAFF2 force-field
library.


DIHE
c3-c3-ce-c2   6    0.000        0.000          3.000      same as X -c3-cc-X
c3-c3-ce-ce   6    0.000        0.000          3.000      same as X -c3-cc-X
hc-c3-cf-cf   1    0.380      180.000         -3.000      same as hc-c3-c2-c2
hc-c3-cf-cf   1    0.000        0.000         -2.000      same as hc-c3-c2-c2
hc-c3-cf-cf   1    1.150        0.000          1.000      same as hc-c3-c2-c2
hc-c3-cf-ce   1    0.380      180.000         -3.000      same as hc-c3-c2-c2
hc-c3-cf-ce   1    0.000        0.000         -2.000      same as hc-c3-c2-c2
hc-c3-cf-ce   1    1.150        0.000          1.000      same as hc-c3-c2-c2
hc-c3-ce-cf   1    0.380      180.000         -3.000      same as hc-c3-c2-c2
hc-c3-ce-cf   1    0.000        0.000         -2.000      same as hc-c3-c2-c2
hc-c3-ce-cf   1    1.150        0.000          1.000      same as hc-c3-c2-c2
hc-c3-ce-ce   1    0.380      180.000         -3.000      same as hc-c3-c2-c2
```

hc-c3-ce-ce	1	0.000	0.000	-2.000	same as hc-c3-c2-c2
hc-c3-ce-ce	1	1.150	0.000	1.000	same as hc-c3-c2-c2
c3-c3-cf-cf	6	0.000	0.000	3.000	same as X -c3-cd-X
c3-c3-cf-c2	6	0.000	0.000	3.000	same as X -c3-cd-X

IMPROPER

c3-c3-c2-ce	1.1	180.0	2.0	Using the default value
c2-c3-ce-ce	1.1	180.0	2.0	Using the default value
ce-cf-ce-ha	1.1	180.0	2.0	Same as X -X -ca-ha
ce-cf-cf-ha	1.1	180.0	2.0	Same as X -X -ca-ha
c3-ce-cf-cf	1.1	180.0	2.0	Using the default value
c3-ce-ce-cf	1.1	180.0	2.0	Using the default value
c2-c3-cf-cf	1.1	180.0	2.0	Using the default value
c3-c3-c2-cf	1.1	180.0	2.0	Using the default value

NONBON
#LJ parameters for the assigned atom types can be found in the AMBER GAFF2 force-field library.

References

1. J. Hall, T. Renger, F. Müh, R. Picorel and E. Krausz, *Biochim. Biophys. Acta Bioenerg.*, 2016, **1857**, 1580-1593.

2. Y. Shibata, S. Nishi, K. Kawakami, J.-R. Shen and T. Renger, *J. Am. Chem. Soc.*, 2013, **135**, 6903-6914.

3. T. Reinot, J. Chen, A. Kell, M. Jassas, K. C. Robben, V. Zazubovich and R. Jankowiak, *Anal. Chem. Insights*, 2016, **11**, 35-48.
